# Decreased SGK1 Expression and Function Contributes to Behavioral Deficits Induced by Traumatic Stress

**DOI:** 10.1371/journal.pbio.1002282

**Published:** 2015-10-27

**Authors:** Pawel Licznerski, Vanja Duric, Mounira Banasr, Kambiz N. Alavian, Kristie T. Ota, Hyo Jung Kang, Elizabeth A. Jonas, Robert Ursano, John H. Krystal, Ronald S. Duman, Traumatic Stress Brain Study Group

**Affiliations:** 1 Department of Psychiatry, Yale University School of Medicine, New Haven, Connecticut, United States of America; 2 Department of Internal Medicine, Section of Endocrinology, Yale University School of Medicine, New Haven, Connecticut, United States of America; 3 Department of Physiology and Pharmacology, Des Moines University, Des Moines, Iowa, United States of America; 4 Division of Brain Sciences, Department of Medicine, Imperial College London, London, United Kingdom; 5 Department of Psychiatry and Center for the Study of Traumatic Stress, Uniformed Services University of the Health Sciences and for the Traumatic Stress Brain Study Group, Bethesda, Maryland, United States of America; 6 Abraham Ribicoff Research Facilities, Connecticut Mental Health Center, New Haven, Connecticut, United States of America; 7 VA National Center for PTSD, VA Connecticut Healthcare System, West Haven, Connecticut, United States of America; 8 Yale-New Haven Hospital, New Haven, Connecticut, United States of America; Emory University, UNITED STATES

## Abstract

Exposure to extreme stress can trigger the development of major depressive disorder (MDD) as well as post-traumatic stress disorder (PTSD). The molecular mechanisms underlying the structural and functional alterations within corticolimbic brain regions, including the prefrontal cortex (PFC) and amygdala of individuals subjected to traumatic stress, remain unknown. In this study, we show that serum and glucocorticoid regulated kinase 1 (*SGK1*) expression is down-regulated in the postmortem PFC of PTSD subjects. Furthermore, we demonstrate that inhibition of SGK1 in the rat medial PFC results in helplessness- and anhedonic-like behaviors in rodent models. These behavioral changes are accompanied by abnormal dendritic spine morphology and synaptic dysfunction. Together, the results are consistent with the possibility that altered SGK1 signaling contributes to the behavioral and morphological phenotypes associated with traumatic stress pathophysiology.

## Abstract 2

Activity of the protein kinase SGK1 is reduced in the prefrontal cortex of individuals with post-traumatic stress disorder (PTSD), and SGK1 inhibition can cause PTSD-related behavioral changes in an animal model.

## Author Summary

Improper functioning of the brain regions known as prefrontal cortex and amygdala is associated with the development of post-traumatic stress disorder. However, little is known about the molecular mechanisms that underlie this condition. We found that the expression of a protein kinase involved in cellular responses to stress, known as serum and glucocorticoid regulated kinase 1 (SGK1), was decreased in the prefrontal cortex of subjects who had died with post-traumatic stress disorder. Furthermore, we found that experimentally decreasing SGK1 function in the prefrontal cortex of rats resulted in behaviors characteristic of traumatic stress, including the unwillingness to avoid discomfort and the inability to experience pleasure. Finally, reduced SGK1 function in neurons affected their basic electrophysiological properties and caused a decrease in the number of dendritic spines—the specialized protrusions of dendrites that receive synaptic inputs.

## Introduction

Major depressive disorder (MDD) and post-traumatic stress disorder (PTSD) are highly comorbid. Approximately 50% of newly diagnosed patients with PTSD meet criteria for MDD [1]. Exposure to traumatic stress is an important factor in the etiology of both, MDD and PTSD. Regardless of the type of trauma, symptoms include increased fear and anxiety, intrusive thoughts and dreams of the trauma, conditioned fear and hyperarousal including increased startle response, avoidance of reminders of the trauma, and emotional numbing and anhedonia [2,3]. Neuroimaging studies in PTSD patients show an impaired function of medial prefrontal cortex (mPFC) [4–6], resulting in decreased inhibitory control of amygdala function that contributes to increased anxiety and fear [7–9]. This general pattern of brain circuit dysfunction also emerges in healthy humans exposed to uncontrollable stress [10]. Decreased responsiveness of the mPFC leads to deficits in the ability to extinguish fear responses, which contributes to the persistence of traumatic memories seen in patients with PTSD [7,8,11].

Inescapable stress, originally recognized as an animal model for depression, produces a syndrome that shares many behavioral, physiologic, and neurochemical features observed in MDD and PTSD [12–15]. In addition, morphological studies conducted in rodent mPFC show that chronic stress causes a reduction of dendrite length and spine density of pyramidal neurons, and these alterations are associated with impairments in mPFC-mediated cognitive tasks [16].

Despite this progress on circuit level analysis of PTSD pathophysiology, there is very little known about the underlying molecular and cellular alterations that cause PTSD. To address this issue, we have established the first PTSD postmortem brain bank with a small cohort of currently available cases *(n* = 6; [five females and one male] and controls *n* = 6; (six females) and have conducted a microarray study of these subjects and matched controls. This has resulted in identification of a number of dysregulated genes in the PFC of PTSD subjects, one of which is serum and stress activated kinase 1 (*SGK1*). Further studies in rodent models demonstrate that disruption of SGK1 signaling results in behavioral and morphological endophenotypes associated with traumatic stress, including helplessness- and anhedonic-like behaviors and decreased spine density in mPFC neurons.

## Results

**Fig 1:**
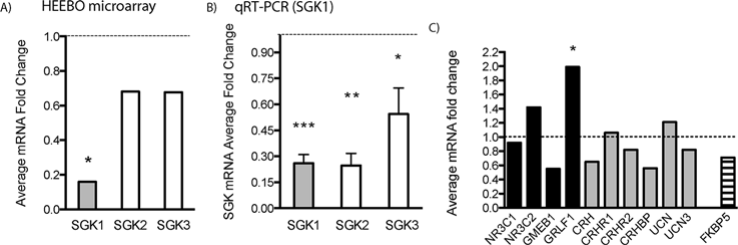
SGK1 expression is significantly decreased in the prefrontal cortex of PTSD patients. (A) Microarray gene expression analysis of postmortem dorsolateral PFC (DLPFC) samples collected from patients with PTSD shows decreased levels of SGK1 mRNA. Microarray analysis demonstrates that levels of SGK2 and SGK3 isoforms are reduced, but these effects were not significant. (B) Real-time qPCR was conducted to verify the microarray findings. Data are expressed as a mean fold change ± standard error of the mean (SEM) (*n* = 6), compared to healthy controls (*n* = 6). [t(10) = 5.966 for SGK1; t(8) = 4.275 for SGK2; t(8) = 2.057 for SGK3, Student’s *t* test, *p < 0.05, **p < 0.01, ***p < 0.001]. (C) Expression levels of stress- and glucocorticoid-regulated genes were also examined. Changes are shown as a mean fold change. Asterisk indicates a significant *p*-value (*p < 0.05, FDR adjusted). For underlying data, see S1 Data. NR3C1, glucocorticoid receptor; NR3C2, mineralocorticoid receptor; GMEB1, glucocorticoid modulatory element binding protein 1; GRLF1, glucocorticoid receptor DNA binding factor 1; CRH, corticotropin-releasing hormone; CRHR1, corticotropin-releasing hormone receptor 1; CRHR2, corticotropin-releasing hormone receptor 2; CRHBP, corticotropin-releasing hormone binding protein; UCN, urocortin; UCN3, stresscopin (urocortin 3); FKBP5, FK506 binding protein 5.

To identify transcriptional alterations that could contribute to traumatic stress in the PFC, we conducted a whole genome array of postmortem PFC samples collected from a small cohort of individuals diagnosed with PTSD and age-matched individuals without psychiatric diagnoses (S1 Table). The microarray study identified 231 down-regulated and 42 up-regulated genes in the PFC of PTSD subjects (S2 Table). One of the most highly dysregulated genes is *SGK1*, which is reported to be induced by corticosteroid hormones and has been implicated in cellular responses to stress [17,18]. SGK1 belongs to the serine/threonine family of protein kinases consisting of three isoforms, *SGK1*, *SGK2* and *SGK3* [18,19]. Our array analysis using the false discovery rate (FDR) method for multiple comparisons [20] shows that SGK1 expression is down-regulated by over 80% in PFC from PTSD subjects relative to the control group (Fig 1A). Levels of *SGK2* and *SGK3* expression were also decreased relative to controls, although the microarray results were not significant (Fig 1A). Quantitative PCR analysis was used to verify the microarray findings and demonstrates that expression levels of *SGK1*, as well as *SGK*2 and *SGK3*, are significantly decreased in the PFC of PTSD subjects (Fig 1B and S3 Table). Several other stress- and glucocorticoid-regulated proteins were also examined, although only the glucocorticoid receptor DNA binding factor 1 (*GRLF1*) was significantly altered in the PFC of PTSD subjects (Fig 1C). Previous studies have reported that FKBP5 levels are decreased [21] or increased [22] in blood or lymphocytes of PTSD patients. In our microarray study, *FKBP5* expression was down-regulated in PTSD samples, but this result did not pass FDR for significance. Out of three *SGK* isoforms, we decided to investigate *SGK1*, as it is the only gene among the three *SGK* family members that is transcriptionally regulated by glucocorticoids [23–25]. In addition, *SGK1* is known to regulate the function of transcription factors, ion channels, and ion carriers and is reported to play a role in cellular and behavioral models of learning and memory [23].

**Fig 2:**
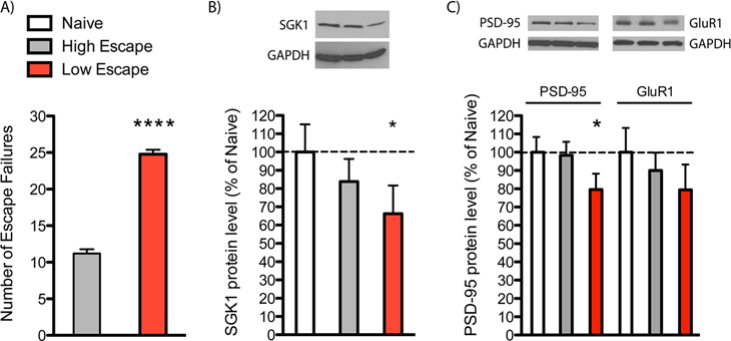
SGK1 expression is altered in learned helplessness. **(**A) Rats were exposed to inescapable shock (day 1), tested in active avoidance (AA) (day 4), and then sacrificed (day 8) as indicated. Animals were separated into low- and high-escape groups according to their performance in AA test. Data are the mean ± SEM [t(12) = 14.39, Student’s *t* test, ****p < 0.0001]. (B) SGK1 and (C) PSD-95 protein levels in dissected PFC are decreased in the low-escape group. Data are mean ± SEM percent change over control group (naive, *n* = 5; high escape, *n* = 5; low escape, *n* = 9), One-way ANOVA with post hoc Bonferroni test: F(2,16) = 3.373 for SGK1 and F(2,16) = 3.704 for PSD-95, * *p* < 0.05. For underlying data, see S1 Data.

To investigate the effects of *SGK1* on rodent behavior, we used a rat inescapable stress paradigm that has been employed in rodent studies of depression but that also models some features of PTSD [23,24]. In this paradigm, exposure to acute foot shock stress results in loss of escape behavior, referred to as helplessness. After a series of inescapable foot shocks on day 1, rats were tested in active avoidance on day 4. Approximately half of the animals failed to escape the foot shocks, consistent with previous reports in outbred animals [25]. These animals are referred to as the low-escape group (>12 or more failures). The remaining animals learned to escape and are referred to as the high-escape group (<12 failures) (Fig 2A). There was a significant decrease in SGK1 protein levels in PFC (dissections included prelimbic, infralimbic, and cingulate PFC) of low-escape rats, compared to naïve, similar to the decrease in PTSD subjects (Fig 2B). We also observed decreased *SGK1* mRNA, but not protein levels in the hippocampus (S1 Fig). As *SGK1* expression is induced by glucocorticoids, we measured protein levels of glucocorticoid receptor (GR) in the PFC and found no significant changes between naïve, high-, and low-escape rats (S2 Fig). We also found that levels of postsynaptic density protein-95 (PSD-95) were significantly lower in the low-escape group compared to naïve or high-escape rats (Fig 2C). Levels of GluR1, an ionotropic glutamate receptor, were also reduced, but not significantly, in the low-escape group (Fig 2C). Plasma corticosterone levels were slightly elevated in high- and low-escape animals compared to control but did not reach significance (S3 Fig).

**Fig 3:**
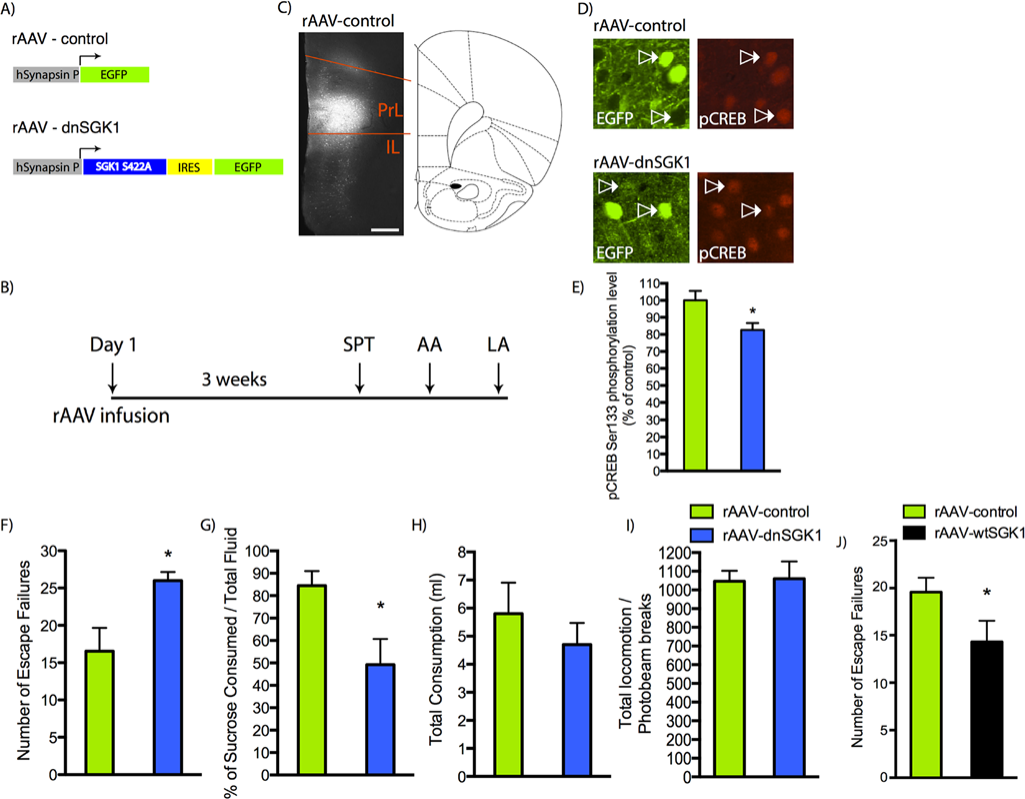
Viral expression of dnSGK1 increases escape failures. (A) rAAV construct used to express dnSGK1/EGFP or EGFP alone (control). ITR, inverted terminal repeats; IRES, internal ribosomal entry site. (B) Experimental timeline for behavioral analysis after bilateral mPFC infusions of rAAV-EGFP, rAAV-dnSGK1, or rAAV-wtSGK1. SPT, sucrose preference test; LA, locomotor activity. (C) Shown is a representative low-power magnification of green fluorescent protein (GFP) expression in the medial PFC after infusion of rAAV-dnSGK1. Expression was observed in the prelimbic (PrL) and infralimbic (IL) regions of the medial PFC. Scale bar = 500 μm. (D) Colocalization of dnSGK1 and phospho-CREB (pCREB) in the prefrontal cortex: pCREB Ser133 immunostaining in rat PFC from animals infused with rAAV-control and rAAV-dnSGK1. (E) Quantification of pCREB-positive cells showed an 18% reduction in the dnSGK1-overexpressing group (rAAV-control, *n* = 16 neurons; rAAV-dnSGK1, *n* = 16 neurons; two rats per group). Data are mean ± SEM percent change over control group. (rAAV-control 100 ± 5.58; rAAV-dnSGK1 82.55 ± 4.09.) (t(30) = 2.094, *p < 0.05, Student’s *t* test.) Effects of rAAV-dnSGK1 infusion into medial PFC on behavior were tested in (F) AA (escape failures), (G) sucrose preference, (H) total fluid consumption, and (I) locomotor activity. Data are shown as mean ± SEM (controls *n* = 11; dnSGK1 *n* = 9). (t(18) = 2.61 for AA and t(18) = 2.795 for SPT, Student’s *t* test, *p < 0.05]. (J) AA (escape failures) for rAAV-wtSGK1 injected rats (controls *n* = 10; wtSGK1 *n* = 10). (t(18) = 1.933, one-tailed Student’s *t* test, *p < 0.05.) For underlying data, see S1 Data.

To determine whether the behavioral differences between the low- and high-escape groups are due to decreased SGK1 activity, we developed an approach to inhibit SGK1 function by viral expression of a dominant negative form of SGK1 (SGK1 S422A, referred to as dnSGK1). Enhanced green fluorescent protein (EGFP) and/or dnSGK1 were expressed under the regulation of the synapsin I promoter using recombinant adeno-associated virus (rAAV) (Fig 3A and S5 Fig). rAAV-dnSGK1 or rAAV-EGFP control viruses were infused into the mPFC of rats, and after 3 wk to allow for optimal viral expression, they were assessed in behavioral models according to the timeline shown in Fig 3B. Expression and location of the infusion were confirmed by analysis of EGFP (Fig 3C). Infusions of rAAV-dnSGK decreased Ser133 phosphorylation of cAMP response element-binding protein (CREB), a direct SGK1 phosphorylation target, demonstrating the functional consequences of dominant negative *SGK* expression (Fig 3D and 3E). Studies in primary cultures demonstrate that dnSGK1 does not influence total CREB levels (S6 Fig), consistent with previous reports [26], indicating that decreased phospho-CREB (pCREB) is due to decreased phosphorylation of CREB, not total CREB levels. In the absence of prior foot shock stress, rats infused with rAAV-dnSGK1 displayed significantly fewer escapes compared to rAAV-EGFP controls (Fig 3F), similar to the low-escape group exposed to foot shock stress. In contrast, infusion of rAAV-overexpressing SGK1 (referred to as wtSGK1) resulted in a higher number of escapes in this paradigm (Fig 3J). rAAV-dnSGK1 rats also displayed significantly decreased preference for a sweetened sucrose solution, an indication of anhedonia (Fig 3G). There was no difference in total fluid consumption (Fig 3H) or locomotor activity (Fig 3I) between groups. These studies demonstrate that inhibition of SGK1 is sufficient to cause helplessness- and anhedonic-like behaviors. Infusions of rAAV-dnSGK1 compared to rAAV-EGFP had no significant effects on behavior in anxiety-based models, including the elevated plus maze, novelty-suppressed feeding, and open field (note that rAAV-EGFP control and rAAV-dnSGK1 rats displayed short center time in the open field, which could occlude any further decrease that would be suggestive of increased anxiety) or forced swim test (S4 Fig).

**Fig 4:**
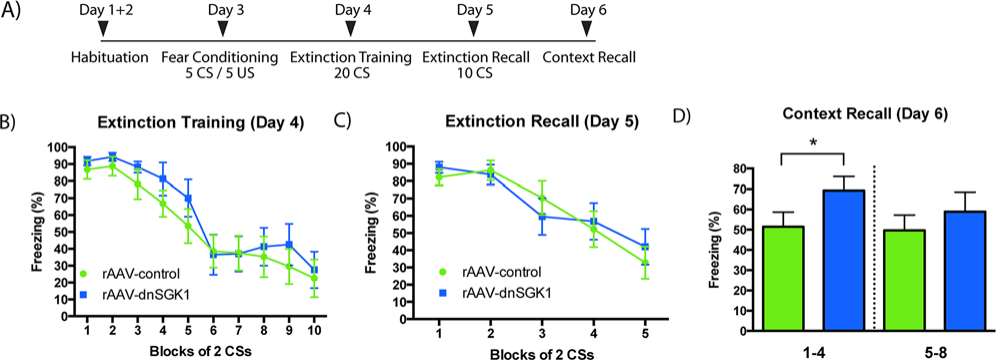
Influence of viral expression of dnSGK1 on contextual memory recall. (A) Rats were infused with rAAV-dnSGK1 or rAAV-EGFP into the mPFC. Four weeks post infusion rats underwent auditory fear conditioning and then extinction training and testing according to the timeline schedule shown. (B) During extinction training, CS-elicited freezing was comparable between the dnSGK1 and EGFP groups (effect for group F(1,18) = 0.7033, *p* = 0.4127; for trial F(9,162) = 23.15, *p <* 0.0001; interaction F(9,162) = 0.3374, *p* = 0.9613, two-way repeated measures ANOVA). (C) During extinction recall, there was no significant difference in freezing between dnSGK1 and controls (effect for group F(1,18) = 0.01975, *p* = 0.8898; for trial F(4,72) = 20.27, *p <* 0.0001; interaction F(4,72) = 0.7638, *p* = 0.5523, two-way repeated measures ANOVA). (D) When reexposed to the original context, rats infused with dnSGK1 showed significantly higher freezing during the first 4 min of testing (t(18) = 1.787, one-tailed Student’s *t* test, *p < 0.05), but not during the second 4-min block. For underlying data, see S1 Data.

To further analyze the role of SGK1 in relation to PTSD, we used fear conditioning and extinction as another model of PTSD-related behavior that can be tested in animals [27,28]. First, rAAV-dnSGK1 or rAAV-EGFP control virus were infused into the mPFC. After 4 wk, rats were subjected to an auditory fear conditioning and extinction paradigm (Fig 4A). Rats were fear conditioned (day 3) and then underwent extinction training (day 4) and recall testing (day 5) in a contextually distinct environment from that used for conditioning. During the extinction training session, there was no significant difference in levels of conditioned stimulus (CS)-elicited freezing between the dnSGK1 and EGFP controls (Fig 4B), indicating a similar rate and magnitude of extinction between both groups. Twenty-four hours later, the rats were returned to the extinction context in order to assess extinction recall (day 5, Fig 4C). The dnSGK1-expressing rats did not show significantly higher levels of overall freezing compared to EGFP controls, indicating that inhibition of SGK1 did not affect the acquisition of extinction or impair the consolidation of extinction learning. Twenty-four hours after the extinction recall session, the animals were reexposed to the context in which they were fear conditioned to examine context-dependent fear (day 6, Fig 4D). Rats infused with rAAV-dnSGK1 displayed higher levels of freezing compared to EGFP controls during the first 4 min of contextual memory recall test (Fig 4D), although there was no effect during the second block of 4 min. Taken together, the results demonstrate that inhibition of SGK1 enhances the memory of contextual cues associated with fear conditioning.

**Fig 5:**
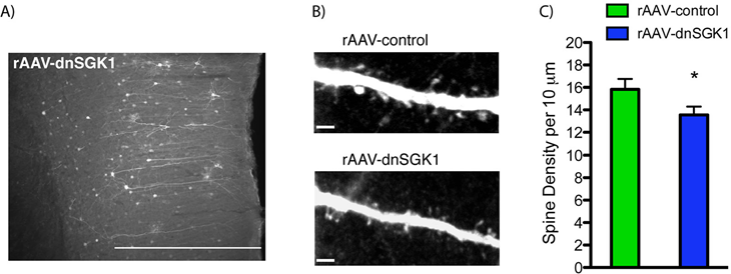
Impact of dnSGK1 overexpression on spine density in the medial PFC. rAAV-dnSGK1was infused into the medial PFC of rats, and 3 wk later, tissue was collected for analysis of spine density. (A) Low-power image of EGFP labeled layer II/III pyramidal neurons. Scale bar = 500 μm. (B) High-power image of representative dendrite segments taken from rAAV-control or rAAV-dnSGK1 showing altered spine number. Scale bar = 4 μm. (C) Spine density analysis of layer II/III pyramidal dendrite segments sampled from rAAV-dnSGK1 injected mPFC (mostly prelimbic cortex) (animals, *n* = 5; branches, *n* = 10, total dendritic length = 347.81 μm) or rAAV-EGFP (animals, *n* = 7; branches, *n* = 14, total dendritic length = 404.46 μm). Data are shown as mean ± SEM compared to the rAAV-EGFP control group (t(22) = 1.777, Student’s *t* test, *p < 0.05). For underlying data, see S1 Data.

**Fig 6:**
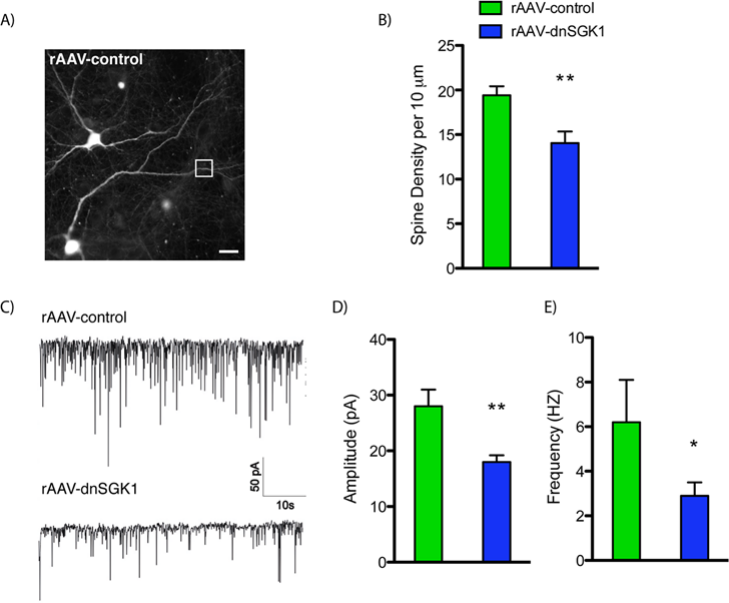
Impact of dnSGK1 overexpression on dendritic spine density and electrophysiological properties in cultured neurons. Primary neuronal cultures were incubated with rAAV-control or rAAV-dnSGK1, and spine density and function were analyzed (14–16 DIV). (A) Representative image of EGFP labeling in primary neurons exposed to control virus. Scale bar = 25 μm. (B) Quantitation of spines density showing a decrease in neurons cultured with dnSGK1 (neurons, *n* = 6; branches, *n* = 7; total dendritic length = 174.36 μm) as compared to EGFP control (neurons, *n* = 5; branches, *n* = 7; total dendritic length = 197.95 μm). Data are shown as mean ± SEM, compared to the control group (t(12) = 3.24, Student’s *t* test, **p < 0.01]. (C) Analysis of spontaneous mEPSCs (representative recording traces) in hippocampal primary neurons (16 DIV) overexpressing dnSGK1 (*n* = 7) showed a decrease in amplitude (D) and frequency (E) of mEPSCs compared to EGFP controls (*n* = 6). Data are shown as mean ± SEM, compared to the control group (t(11) = 4.335, **p < 0.01 for the amplitude; t(11) = 2.738, *p < 0.05 for the frequency, Student’s *t* test). For underlying data, see S1 Data.

To examine the cellular mechanisms underlying these behavioral effects of SGK1 inhibition, levels of synaptic density and function were analyzed in rat mPFC and in primary neuronal cultures. In our initial studies of *SGK1* expression in the learned helplessness model, we observed decreased levels of the synaptic protein PSD-95 and a trend for decreased levels of the glutamate receptor GluR1 in the low-escape group (Fig 2C), suggesting that the number of functional spine synapses may be altered in low-escape rats. To directly test this possibility, we examined the influence of SGK1 inhibition on dendritic spines of layer II/III pyramidal neurons in mPFC. Infusion of rAAV-dnSGK1 into the mPFC significantly decreased the density of spines on the dendrites of layer II/III neurons compared to animals receiving control virus (Fig 5A–5C). This indicates that SGK1 is necessary for maintaining the structural integrity of dendritic spines in the PFC. Studies in primary neuronal cultures (14–16 days in vitro [DIV]) further demonstrate that rAAV-dnSGK1 significantly decreases the number of spines on apical dendrites of neurons (Fig 6A and 6B) as well as the function of spine synapses, demonstrated by a reduction of the amplitude (43%) and frequency (36%) of miniature evoked-postsynaptic currents (mEPSCs) (Fig 6C–6E). These in vivo and in vitro findings suggest that the reduction in spine number and function associated with decreased *SGK1* expression could contribute to the structural changes of PFC in PTSD patients [29,30] and to the loss of PFC inhibitory control of connected target regions [27,28].

## Discussion

Whole genome microarray analysis demonstrates altered expression of a number of genes in postmortem PFC of PTSD subjects. Based on magnitude of change and potential functional impact in response to stress and trauma, we chose *SGK1* as a candidate that could contribute to the pathophysiology of PTSD. A recent report describes the role of SGK1 in cortisol-induced reduction of progenitor cell proliferation in the hippocampus and enhancing GR function [31], both of which would be relevant to depression. However, in the PFC, *SGK1* expression is not reported to be altered in MDD [32] subjects, indicating possible specificity for PTSD. In addition, the reduction of *SGK1* expression in animals that display decreased escape behavior in the learned helplessness model is consistent with the possible role of *SGK1* in traumatic stress. However, because of the small cohort of PTSD postmortem subjects (five females and one male) that were analyzed, this finding must be replicated with additional PTSD subjects and better representation of both sexes when samples become available to confirm a role of *SGK1* in PTSD. In particular, comparison between PTSD subjects versus those exposed to trauma but with no PTSD diagnosis would be of particular interest, as this comparison would be the most informative in identifying key genes involved in the behavioral deficits observed in PTSD patients.

The results also demonstrate that inhibition of SGK1 in the mPFC causes behavioral deficits characteristic of traumatic stress. We used learned helplessness, a paradigm often employed for studies of depression, because the behavioral deficits are induced by traumatic stress and because helplessness is often associated with PTSD [33].

Given the high comorbidity between MDD and PTSD and the fact that exposure to trauma is a risk factor for MDD as well as PTSD, it has been suggested that pathways mediating learned helplessness are activated in multiple disorders induced by uncontrollable stress, including PTSD and MDD [34].

Supporting the utility of this model, we found that a subgroup of rats that showed decreased avoidance (i.e., helpless-like behavior) also expressed lower levels of *SGK1* mRNA and protein, similar to the deficits observed in PTSD postmortem subjects. We directly tested the role of SGK1 in helpless-like behavior using a viral vector and found that overexpression of a dominant negative form of SGK1 in the mPFC reproduced the deficit in active avoidance observed in animals exposed to inescapable stress. In contrast, overexpression of wtSGK1 in mPFC increased the number of escapes in the AA paradigm, compared to control group. We also found that expression of dnSGK1 in the mPFC caused anhedonic-like behavior (i.e., decreased preference for a sweet solution). These studies demonstrate that inhibition of SGK1 is sufficient to cause helplessness- and anhedonic-like behaviors reported in PTSD [33].

Another hallmark of PTSD is the inability to extinguish or inhibit maladaptive fear responses to stimuli previously associated with a traumatic event, even when there is no possibility for recurrence of the trauma. We found that overexpression of dnSGK1 in the PFC enhanced freezing in a context in which the rats had been fear conditioned. These findings demonstrate the functional impact of SGK1 for proper processing of context-associated fear memory. Recent studies showed the importance of mPFC not only for contextual fear conditioning but also for expression of contextual fear [35,36]. These processes require mPFC activity and N-methyl-D-aspartate (NMDA) receptor activation [37,38]. Our findings are consistent with a previous report demonstrating that impaired fear inhibition is a specific marker of PTSD [39].

We also examined the possibility that inhibition of SGK1 causes cellular deficits that could underlie the behavioral changes that were observed. SGK1 is reported to influence the morphology of oligodendrocytes in mouse corpus callosum in response to stress [40]. We found that viral expression of dnSGK1 in the mPFC significantly decreased the density of spines on layer II/III pyramidal neurons compared to controls. Studies in primary neuronal cultures (hippocampus) showed that inhibition of SGK1 produced a similar decrease in the density as well as function of spine synapses (i.e., decreased mEPSCs), although these findings must be confirmed in primary cortical cultures. Together these findings indicate that normal levels of SGK1 are necessary to maintain the structural and functional integrity of spine synapses in the PFC. Moreover, the reduction in spine number and function associated with decreased *SGK1* expression could contribute to the structural changes of PFC in PTSD patients [29,30] and to the loss of PFC inhibitory control of amygdala that is required for the extinction of fear memory in animals and humans [27,28]. Further studies will be required to determine the functional impact of decreased SGK1 in vivo and to determine the underlying mechanisms by which SGK1 influences synaptic density and function, such as regulation of the insertion of synaptic glutamate receptors. For example, SGK1 is reported to inhibit stress-induced potentiation of glutamatergic transmission through regulation of NMDA and AMPA, α-amino-3-hydroxy-5-methyl-4-isoxazolepropionic acid (AMPA) receptor trafficking, as well as to produce more long-term effects via regulation of transcription factors [17,41,42].

In summary, the results of this study demonstrate that SGK1 expression is decreased in the postmortem PFC of a small cohort of PTSD subjects, a finding that must be confirmed in additional PTSD subjects. In addition, studies are currently underway to examine levels of SGK1 in the blood cells of PTSD patients as well as to test for association of SGK1 polymorphisms in PTSD. It is possible that SGK1 may serve as a potential clinical biomarker for PTSD, as recently described for pituitary adenylate cyclase-activating polypeptide (PACAP) [43], which was also shown to contribute to behavioral changes observed in LH [44]. Finally, identification of downstream targets of SGK1 could contribute to a better understanding of the molecular mechanisms underlying the observed behavioral deficits and provide targets for novel PTSD therapeutic medications.

## Materials and Methods

### Ethics Statement

Postmortem brain samples were obtained from the Stanley Medical Group Brain bank, which was responsible for consent from next of kin. All animal procedures were in accordance with United States National Institutes of Health standards and approved by the Yale University Institutional Animal Care and Use Committee (Protocol number: Yale IACUC #2014–07235).

### Human Subjects

The study includes total RNA samples obtained postmortem from six patients diagnosed with PTSD (using DMS-IV criteria) and six age-matched healthy subjects.

### Microarray Analysis

Brains from patients diagnosed with PTSD were matched to brains from healthy subjects based on sex, race, and age. Human Exonic Evidence-Based Oligonucleotide (HEEBO) microarrays (Microarrays, Nashville, Tennessee) were used to analyze changes in gene expression within the PFC. Four μg of total RNA (RIN > 5.0, A260/280 > 2.0) from PTSD (*n* = 6) and matched control human samples (*n* = 6) were reverse transcribed into cDNA and indirectly labeled with highly sensitive fluorescent technology (Genisphere, Hatfield, Pennsylvania). The whole genome expression analysis was performed using two-channel MI Ready microarrays (Microarray, Huntsville, Alabama). Arrays contained 70-mer oligonucleotide probes from HEEBO. First, cDNA was applied on the array chip overnight to allow hybridization. Next, chips went through a series of washes to minimize nonspecific binding and then were labeled with fluorescent Cy3 and Cy5 dendrimers. After posthybridization washes, the array chips were scanned using a GenePix scanner (Axon Instruments, Union City, California), and image analysis was performed using GenePix Pro 6.0 software (Axon Instruments). Statistical expression analysis of microarray image files was performed using GeneSpring GX 7.3.1 software (Silicon Genetics, Redwood City, California). One sample from the control group had to be excluded from the analysis because of poor hybridization. Raw values were normalized to both control channel values (per spot) and to positive control genes (per chip). The level of regulation for an individual gene was calculated by taking the log ratio of the median experimental channel signal to the median control channel signal. Down-regulated genes were identified by an average expression ratio of <0.77-fold, while up-regulated genes were defined as having an average expression ratio of >1.3-fold. Statistical analysis was performed by an unpaired *t*-test without using a cross gene error model within the GeneSpring software. Statistical significance was considered to be *p* ≤ 0.05 with Benjamini and Hochberg FDR as the correction for multiple testing.

### Quantitative Real-Time PCR (qPCR)

Five hundred ng of total RNA extracted from human PFC tissue (six PTSD patients and six control subjects) was reverse transcribed into cDNA using oligo-dT primers (Genisphere, Hatfield, Pennsylvania). Real-time PCR (qPCR) was performed utilizing a hot-start SYBR Green method with an ABI 7900 instrument (Applied Biosystems, Foster City, California) with the following conditions: 2 s at 94°C (denaturation), 30 s at 60°C (annealing), and 30 s at 72°C (elongation), for 40 cycles, using a Quantitect SYBR Green PCR kit (Qiagen, Valencia, California). All primers were designed using Primer3 v. 0.4.0 software (http://bioinfo.ut.ee/primer3-0.4.0/primer3/; Whitehead Institute for Biomedical Research, Cambridge, Massachusetts), and their specificity was verified using nucleotide blast software (BLAST Interface, NCBI). SGK1 gene fold changes in PTSD subjects versus those in controls were determined using ΔΔCt (Ct = cycle number at threshold) analytical method with normalization against housekeeping genes cyclophilin and GAPDH. The sequences of qPCR primers used are as follows: SGK1 forward 5ʹ- cgccggagtatctcgcacctg-3ʹ and reverse 5ʹ-gcggcaggccatacagcatc-3ʹ; cyclophilin forward 5ʹ-caaatcagaatgggacaggtggag-3ʹ and reverse 5ʹ-gtttgtgttgcggcctgcatttg-3ʹ; GAPDH forward 5ʹ-cgggaaactgtggcgtgatgg-3ʹ and reverse 5ʹ-gccagtgagcttcccgttcagc-3ʹ.

### Animals and Behavioral Experiments

Male Sprague-Dawley rats (Charles River Laboratories International) were used for all experiments. All animal procedures were in accordance with US National Institutes of Health standards and approved by the Yale University Institutional Animal Care and Use Committee. For the inescapable foot shock (IFS) and active avoidance (AA) paradigms for rAAV-wtSGK1, rats were habituated to the chamber for 5 min and then subjected to 60 randomized 0.8 mA foot shocks. Three days after IFS or 3 wk after PFC virus infusion, animals were tested in the AA paradigm. The results are expressed as the number of escape failures or the number of times that the animal did not terminate the foot shock by making the appropriate crosses. For the SPT, rats were habituated to 1% sucrose solution (Sigma, St. Louis, Missouri) for 48 h on day 21 post-PFC infusion. The test was performed for 1 h after 4 h deprivation in the presence of two bottles containing 1% sucrose solution and tap water as previously described [45]. The amounts of sucrose solution or water were measured, and the percent sucrose preference was determined.

#### Novelty suppressed feeding

A novelty suppressed feeding test was conducted as previously described [46]. Briefly, rats were food deprived overnight and, on the test day, placed in an open field (76.5 cm × 76.5 cm × 40 cm, Plexiglas) with food pellets in the center. The animals were given 15 min to approach the food and eat. The test was stopped as soon as the animal took the first bite. The latency to eat was recorded in minutes. Home cage food intake was also measured as a control (results are presented in S4 Fig).

#### Elevated plus maze

The elevated plus maze consisted of two open arms (50 × 10 cm) and two closed arms (50 × 10 × 40 cm) arranged such that the two open arms were opposite to each other. The maze was elevated to a height of 50 cm. Rats were placed in the center of the maze facing an open arm and were allowed to explore the maze for 10 min. The total time spent in each arm as well as the number of entries into each arm was recorded (results are presented in S4 Fig).

#### Open field

Open field test was performed in an open field (76.5 cm × 76.5 cm × 40 cm, Plexiglas). Each rat was allowed to freely explore the open field for 10 min. Anxiety-like behavior was assessed as the time spent at the periphery of the open field over a 5-min period (results are presented in S4 Fig).

#### Forced swim test

A forced swim test (FST) was conducted as previously described [47]. Animals were exposed to preswim on day 1; on the next day, rats were placed in a clear cylinder with water (24 ± 1°C, 45-cm depth) for 15 min and scored for the duration of immobility. Immobility was defined as floating or remaining motionless without leaning against the wall of the cylinder (results are presented in S4 Fig).

#### Locomotor activity

Ambulatory locomotor activity was recorded for 30 min using automated cage activity meters equipped with photocells (Digiscan animal activity monitor, Omnitech Electronics, Columbus, Ohio) and measured as a number of beam breaks (results are presented in S4 Fig).

#### Western blot analysis

Fresh rat PFC and hippocampal tissue was homogenized in lysis buffer (25 mM HEPES, 300 mM NaCl pH 7.4, 2% Triton-100) containing proteinase inhibitors (Roche, Indianapolis, Indiana) and phosphatase inhibitors (10 mM NaF, 1 mM NaVO3). The protein concentration was measured using a BCA kit (Pierce, Rockford, Illinois). Then, protein samples were electrophoretically separated on an SDS-PAGE gel (10% Tris-HCl; Bio-Rad, Hercules, California) and transferred overnight to nitrocellulose membranes (0.2 μm pores; Millipore, Bedford, Massachusetts). The membranes were incubated in 5% dry, nonfat milk blocking solution (Tris-buffered saline [TBS], 0.1% Tween 20) for 1 h and then incubated at 4°C overnight with primary anti-SGK1 (1:400, Novagen), anti-PSD-95 (1:1000, Invitrogen), anti-GluR1 (1:1000, Abcam), anti-GR (1:1000, Santa Cruz), and anti-GAPDH (1:10000, Advanced Immunochemical) in 1% dry nonfat milk TBST. After 3 x 15 min washes membranes were incubated for 1 h with secondary, horseradish peroxidase (HRP) conjugated antibodies (1:10000, Vector Laboratories, Burlingame, California). Following 3 x 15 min washes in TBST, blots were developed using a chemilluminescence kit (Pierce, Rockford, Illinois) for 5 min and exposed onto HyBlot CL autoradiography film (Denville Scientific, Metuchen, New Jersey). Developed blots were scanned and analyzed using ImageJ software (Scion Corp, Frederick, Maryland).

For in vitro studies, E19 rat hippocampal or cortical primary cultures lysates were prepared in the same way as described above. After SDS-PAGE, nitrocellulose membranes were incubated with anti-CREB (1:1000, Cell Signaling) antibody at 4°C overnight.

### Immunohistochemistry for pCREB

Sections of PFC prepared from perfused (4% paraformaldehyde) brains from rAAV-EGFP and rAAV-dnSGK1 infused rats were blocked in 5% NGS (normal goat serum) for 1 h and incubated overnight at 4°C with anti-pCREB Ser133 (1:800, Upstate) and anti-GFP (Aves Labs) antibodies. After 3 x 15 min washes, the slices were incubated for 2 h with secondary Alexa Fluor 488 and 546-conjugated antibodies (Jacson ImmunoResearch and Invitrogen, respectively), and confocal images were collected. For quantitation of pCREB levels, a region of interest (ROI = GFP positive neurons) was selected using the ImageJ program (ROI manager), first on the GFP image (green channel). The same ROI mask was then applied to the corresponding pCREB image (red channel) for quantitation of pCREB signal intensity. Measured intensity values were corrected for the background nonspecific autofluorescence.

### Construction, Preparation, and Infusion of Recombinant AAV

The rat SGK1 S422A cDNA (dnSGK1), followed by the IRES-EGFP expression cassette, was cloned into an AAV2 backbone containing human Synapsin I promoter. The wtSGK1 cDNA was cloned into AAV2 backbone under the control of Synapsin I promoter. The control rAAV contained Synapsin IRES-EGFP expression cassette. Recombinant AAV2/1 viral stocks were generated as described previously [48]. Briefly, HEK293 cells were transfected using Perfectin reagent (Genlantis, San Diego, California) with AAV *cis* plasmid together with pDp1 and pDp2 helper plasmids. At post-transfection day 3, cell pellets were collected and lysed in three freeze-thaw cycles in dry ice/ethanol bath. Next, collected lysates were filtered using 0.4 μm gauge filters and applied to HiTrap heparine HP affinity columns (GE Healthcare Bio-Sciences AB, Uppsala, Sweden) for AAV purification. Viral titers were determined in rat hippocampal primary cultures. For PFC virus infusions, rats were anesthetized with xylazine (6 mg/kg, IM Lloyd laboratories, Shenandoar, Iowa) and ketamine (80 mg/kg i.m., Fort Dodge Animal Health, Overland Park, Kansas). The virus was delivered bilaterally at +3.5 mm anterior-posterior, ±0.5 mm lateral, and −4 mm ventral from the bregma [49]. Each PFC hemisphere was infused with 3 μl of titer adjusted viral stocks at a rate of 0.2 μl per minute. Behavioral tests were performed 3 wk after virus infusion.

### Auditory Fear Conditioning and Extinction

Four identical conditioning chambers were used in all experiments (30 x 20 x 25 cm; MED-Associates). Fear conditioning and extinction testing occurred in two different contexts. The context was cleaned between animals using a dilute 0.9% acetic acid solution. For extinction training and testing (in a contextually distinct environment), the appearance of the chambers was modified, and in addition, the extinction context was further changed by cleaning the chambers with a 1% almond-scented solution. On days 1 and 2, the rats were habituated to the conditioning chambers for 10-min each. On day 3, the rats were acclimated to the chambers for 3 min. Following this, five auditory CS tones (20 s, 75 dB, 1 kHz) were presented that coterminated with foot shocks (1 s, 1.3 mA). The intertone interval ranged between 60 and 120 s (mean = 78 s). On day 4, the rats underwent extinction training in a different context. After 3 min, the rats received 20 presentations of the CS alone (no US; 60 s intertone interval). Defensive freezing, which was defined as the absence of observable movement except those necessary for respiration, was scored every 3 s during presentation of the tones, and values were averaged across two trials, resulting in ten individual CS blocks for analysis. On day 5, an extinction test was performed in which the rats were returned to extinction context and received ten additional presentation of the CS alone. Again, freezing during the tone was scored every 3 s and averaged across two trials resulting in five individual CS blocks for analysis. Finally, on day 6, the rats were returned to conditioning context and an 8-min extinction test was performed, at which time no tones or foot shocks were delivered. During the context recall test, freezing levels was scored every 8 s during the entire 8-min test. Recordings were scored by an observer blind to the rats’ treatment history.

### Immunocytochemistry and Spine Density Analysis

Brains were perfused with 4% paraformaldehyde and sectioned using a Vibrotome (Leica). 60-μm brain sections were blocked in 5% NGS for 1 h and incubated overnight at RT with chicken anti-GFP primary antibody (1:400, Aves Labs). After 3 x 15 min washes, slices were incubated for 2 h with secondary anti-chicken Alexa Fluor 488-conjugated antibody (Jacson ImmunoResearch). Confocal z-stacks of dendrites (distal tuft branches) were collected using Olympus Fluoview confocal microscope with a 100x objective with constant laser intensity. Total branch length and spine density were determined using Volocity software (Improvision) in 3D restored confocal z-stacks (1024 x 1024 resolution) that went through deconvolution to remove noise pixels. The analysis was performed in the blind fashion, and the spines were counted by hand.

### In Situ Hybridization

In situ hybridization was conducted according to standard previously published protocol used in our laboratory [50]. (^35^S)-radiolabeled, SGK1 specific riboprobes were generated by an in vitro transcription reaction using a PCR product-derived template and MAXIscript kit T7 polymerase (Ambion, Austin, Texas). For generation of the probe template, the following primers (forward and reverse) were used in PCR reaction:

rSGK1 L, 5ʹ-cagagcgcaatgttctgttgaag -3ʹ;

rSGK1 L, 5ʹ-CCAAGCCTTCTAATACGACTCACTATAGGGAGAgtcccattgtgctcgatgttctc-3ʹ

The optical density was quantified within three regions of the hippocampus (CA1, CA3, and DG) using ImageJ (NIH). Using the manufacturer’s calibration scale, specific labeling was determined by subtracting nonspecific binding from total binding.

#### ELISA

Serum was extracted and analyzed using ELISA assay for corticosterone levels according to the manufacturer’s specifications (Assay Designs, Ann Arbor, Michigan).

### Hippocampal and Cortical Primary Cultures

E(18) hippocampal and cortical primary cultures were prepared from Sprague Dawley rat tissue as previously described [51,52] and cultured for 2–3 wk in Neurobasal medium supplemented with B27 (Invitrogen).

### Electrophysiology

The protocol for measuring the spontaneous postsynaptic currents has been described previously [53]. Briefly, the currents were recorded at −70 mV by whole-cell patch-clamp at room temperature (~25°C). Recording electrodes contained (in mM) 120 potassium gluconate, 8 NaCl, 0.5 EGTA, 10 Hepes, and 2 MgATP (pH 7.3). The external solution contained 150 NaCl, 5 KCl, 2.5 CaCl2, 5.5 Hepes-acid, 4.5 Hepes-Na, 10 glucose, and 0.1 tetrodotoxin (pH 7.3). Hippocampal neurons (DIV 16–18) were perfused continuously over the cells, and the inward currents were recorded at resting potential, using Clampex 8, and the data were analyzed, using pClamp 10.2 (Molecular Devices).

## Supporting Information

S1 Data Excel file containing raw data for Figures: Figs 1A–1C, 2A–2C, 3E–3J, 4B–4D, 5C, 6B, 6D, 6E, S1A, S1B, S2B, S3, S4B–S4F, S5 and S6 Figs. (XLSX)

S1 Fig Expression of SGK1 in the hippocampus of rats exposed to learned helplessness. (A) Representative autoradiographs and quantitative analysis of hippocampal SGK1 mRNA levels determined by in situ hybridization in high- and low-escape rats. SGK1 mRNA levels in the CA1 and CA3 pyramidal cell layers and the dentate gyrus granule cell layer were determined. (One-way ANOVA with post hoc Bonferroni test [F(2,16) = 6,014 for CA1, F(2,16) = 3,214 for CA3, and F(2,16) = 7,131 for DG, *p < 0.05). (B) Western blot analysis of SGK1 protein levels in rat whole hippocampal lystates prepared from high- and low-escape rats (naïve, *n* = 5; high escape, *n* = 5; low escape, *n* = 9).
(TIF)

S2 Fig Levels of glucocorticoid receptor (GR) in the prefrontal cortex of rats exposed to learned helplessness. (A) Representative western blot and quantitative analysis of SGK1 protein levels in high- and low-escape rats (naïve, *n* = 5; high escape, *n* = 5; low escape, *n* = 9).
(TIF)

S3 Fig Serum corticosterone levels in rats exposed to learned helplessness. Rats were exposed to inescapable shock (day 1), tested in AA (day 4), and then sacrificed and blood samples were collected (day 8) (naïve, *n* = 5; high escape *n* = 5; low escape *n* = 9).
(TIF)

S4 Fig Influence of dnSGK1 on anxiety-related behaviors. (A) Behavioral evaluation after bilateral PFC infusions of rAAV-EGFP (*n* = 8) or rAAV-dnSGK1 (*n* = 10). Rats were tested in several different anxiety-based animal models, including (B) novelty suppressed feeding (NSF), (C) elevated plus maze (EPM), (D) open field test (OFT), as well as a model of behavioral despair (E), the forced swim test (FST). (F) There was no difference in the locomotor activity between the groups (LA).
(TIF)

S5 Fig Western blot analysis of (A) dnSGK1 and (B) wtSGK1 overexpression using rAAV in rat hippocampal primary cultures. (TIF)

S6 Fig Western blot analysis of total CREB levels in rat cortical primary cultures treated with rAAV-control and rAAV-dnSGK1. Experiment was replicated three times.
(TIF)

S1 Table Case demographics of PTSD patients and matched healthy control subjects. F, female; M, male; C, Caucasian; H, Hispanic; PMI, postmortem interval (hours); RIN, RNA integrity number.
(DOCX)

S2 Table Whole-genome expression analysis of human postmortem PFC. Complete list of up- and down-regulated genes after FDR adjustment for *p*-values. Healthy controls, *n* = 5; PTSD patients, *n* = 6.
(DOCX)

S3 Table Real-time qPCR validation of microarray results for five additional genes. Healthy controls, *n* = 5; PTSD patients, *n* = 6. For the microarray, asterisk indicates significant *p*-value (*p < 0.05, FDR adjusted). Real-time qPCR for FGFR2 t(10) = 3.272, Student’s *t* test, **p < 0.01.
(DOCX)

## Abbreviations

AA: active avoidance
AMPA: α-amino-3-hydroxy-5-methyl-4-isoxazolepropionic acid;
CREB: cAMP response element-binding protein
CS: conditioned stimulus
DIV: days in vitro
DLPFC: dorsolateral PFC
dnSGK1: dominant negative form of SGK1
EGFP: enhanced green fluorescent protein
FDR: false discovery rate
FST: forced swim test
GFP: green fluorescent protein
GR: glucocorticoid receptor
HEEBO: Human Exonic Evidence-Based Oligonucleotide
HRP: horseradish peroxidase
IFS: inescapable foot shock
IM: intramuscular
IRES: internal ribosomal entry site
ITR: inverted terminal repeats
LA: locomotor activity
LH: learned helplessness
mPFC: medial prefrontal cortex
MDD: major depressive disorder
mEPSC: miniature excitatory postsynaptic current
NGS: normal goat serum
NMDA: N-methyl-D-aspartate
PACAP: pituitary adenylate cyclase-activating polypeptide
pCREB: phospho-CREB
PFC: prefrontal cortex
PSD-95: postsynaptic density protein-95
PTSD: post-traumatic stress disorder
qPCR: quantitative PCR
rAAV: recombinant adeno-associated virus
ROI: region of interest
SEM: standard error of the mean
SGK1: serum and glucocorticoid regulated kinase 1
SPT: sucrose preference test
TBS: Tris-buffered saline
wk: weeks
