# Supplementary figures and images for: Decreased SGK1 Expression and Function Contributes to Behavioral Deficits Induced by Traumatic Stress
Source: PLoS Biol. 2015 Oct 27;13(10):e1002282. doi: 10.1371/journal.pbio.1002282 (PMC4623974; doi:10.1371/journal.pbio.1002282)

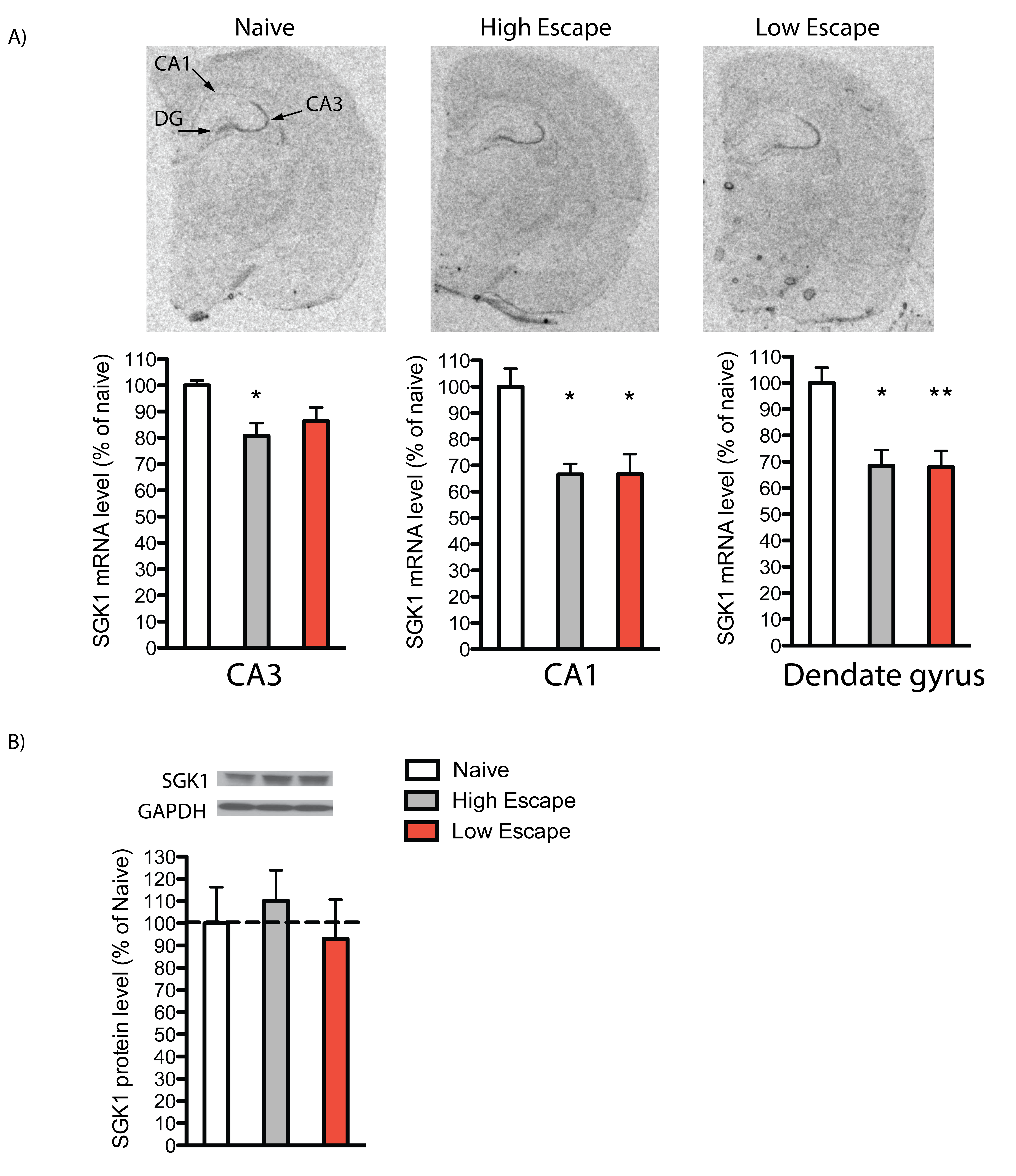

Supplement: S1 Fig — (A) Representative autoradiographs and quantitative analysis of hippocampal SGK1 mRNA levels determined by in situ hybridization in high- and low-escape rats. SGK1 mRNA levels in the CA1 and CA3 pyramidal cell layers and the dentate gyrus granule cell layer were determined. (One-way ANOVA with post hoc Bonferroni test [F(2,16) = 6,014 for CA1, F(2,16) = 3,214 for CA3, and F(2,16) = 7,131 for DG, *p < 0.05). (B) Western blot analysis of SGK1 protein levels in rat whole hippocampal lystates prepared from high- and low-escape rats (naïve, n = 5; high escape, n = 5; low escape, n = 9). (TIF) [file pbio.1002282.s002.tif]

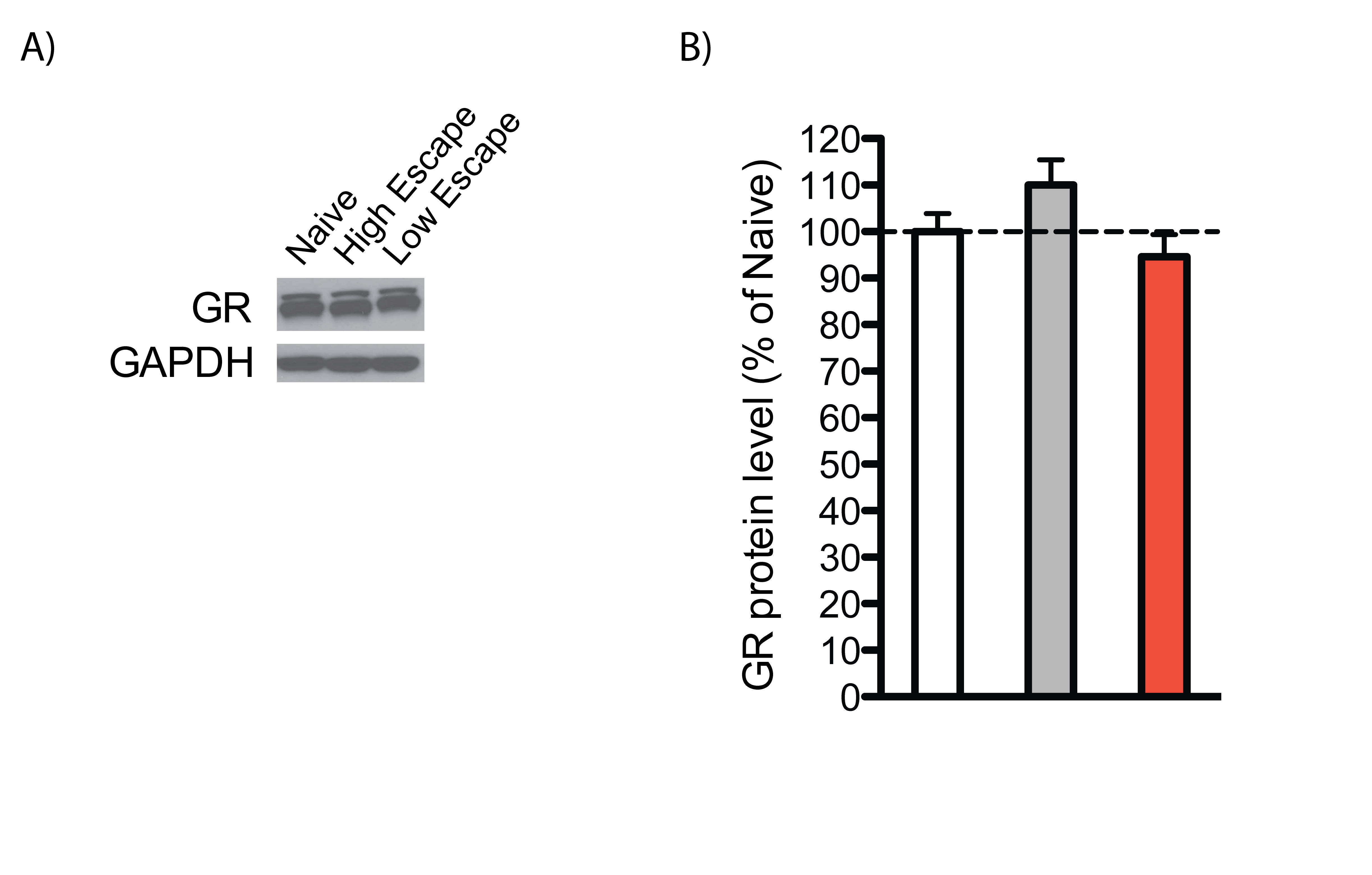

Supplement: S2 Fig — (A) Representative western blot and quantitative analysis of SGK1 protein levels in high- and low-escape rats (naïve, n = 5; high escape, n = 5; low escape, n = 9). (TIF) [file pbio.1002282.s003.tif]

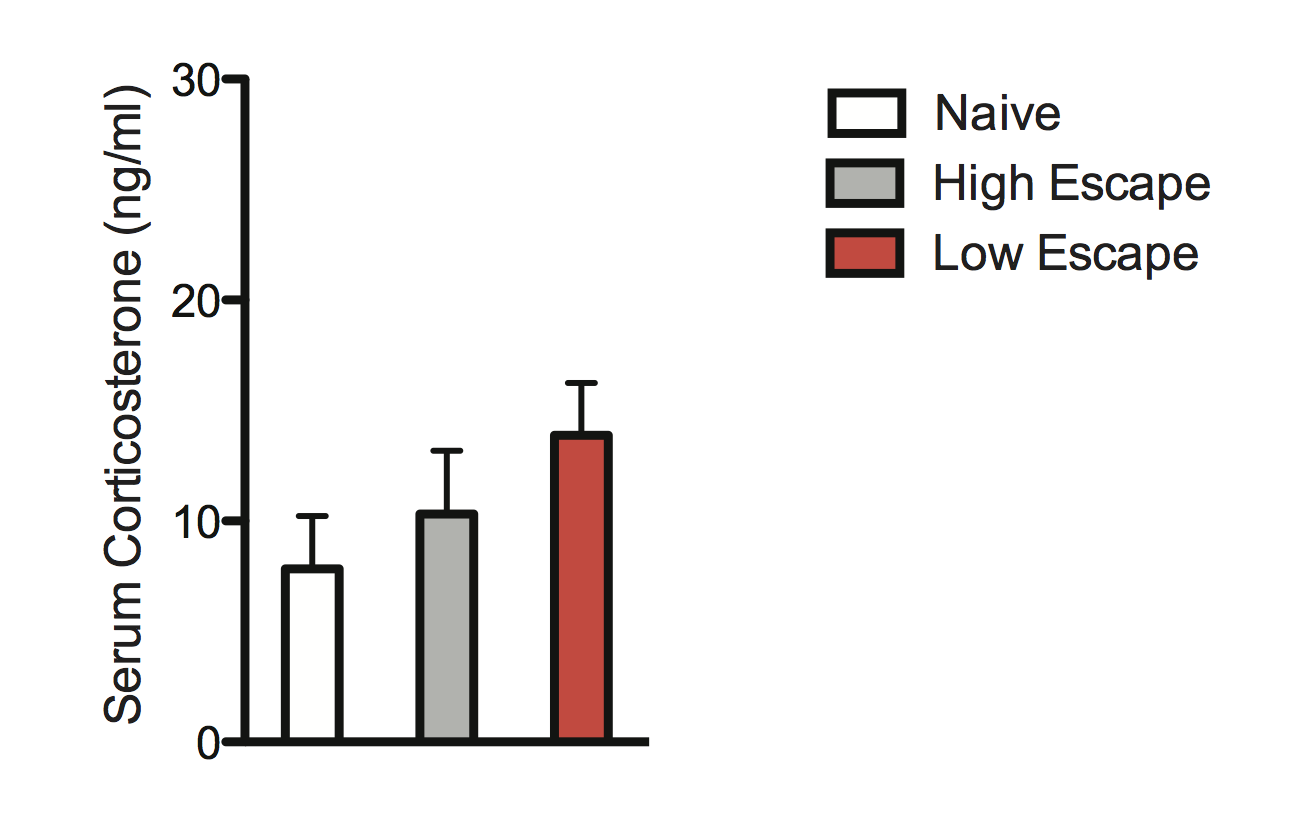

Supplement: S3 Fig — Rats were exposed to inescapable shock (day 1), tested in AA (day 4), and then sacrificed and blood samples were collected (day 8) (naïve, n = 5; high escape n = 5; low escape n = 9). (TIF) [file pbio.1002282.s004.tif]

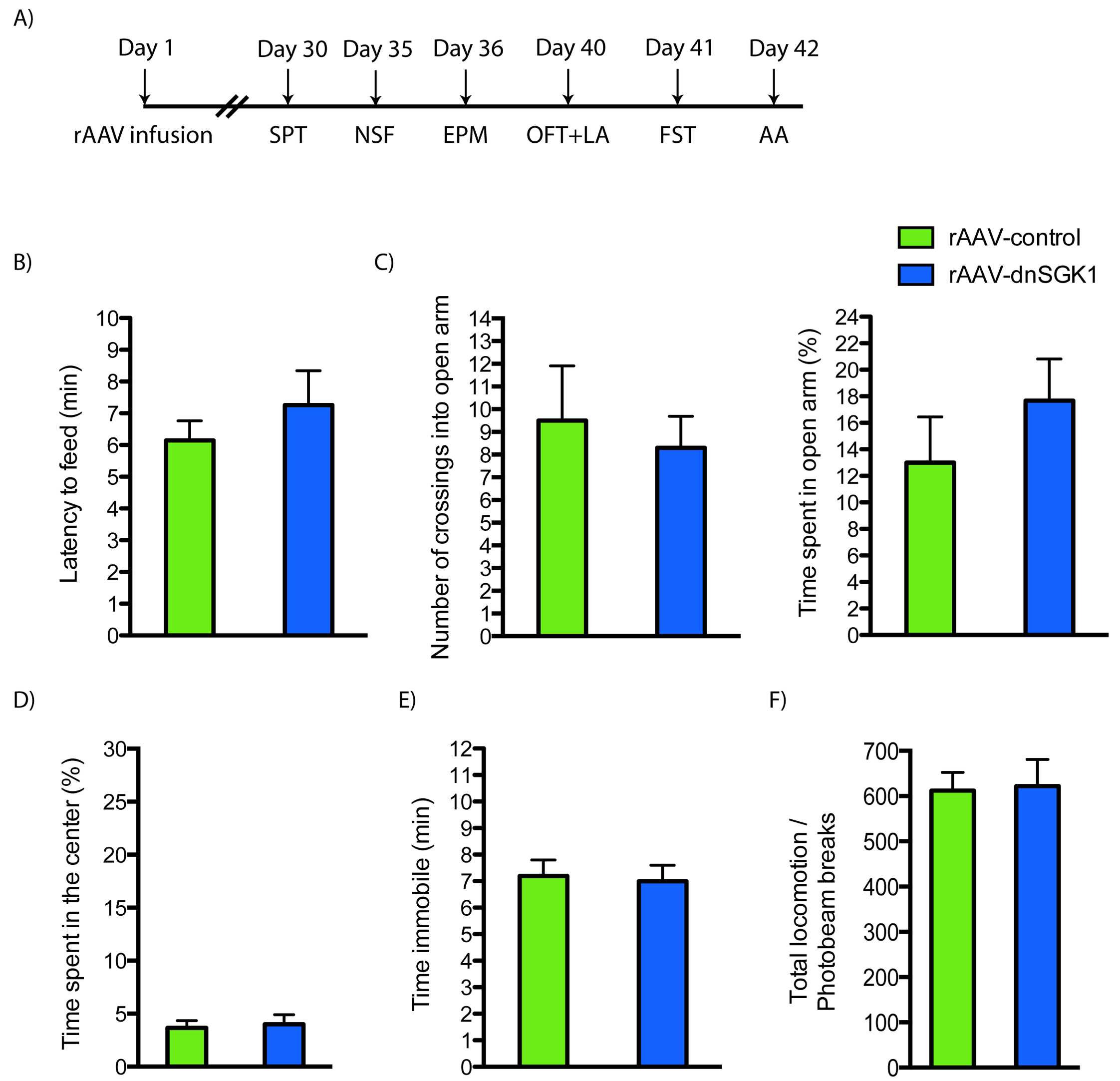

Supplement: S4 Fig — (A) Behavioral evaluation after bilateral PFC infusions of rAAV-EGFP (n = 8) or rAAV-dnSGK1 (n = 10). Rats were tested in several different anxiety-based animal models, including (B) novelty suppressed feeding (NSF), (C) elevated plus maze (EPM), (D) open field test (OFT), as well as a model of behavioral despair (E), the forced swim test (FST). (F) There was no difference in the locomotor activity between the groups (LA). (TIF) [file pbio.1002282.s005.tif]

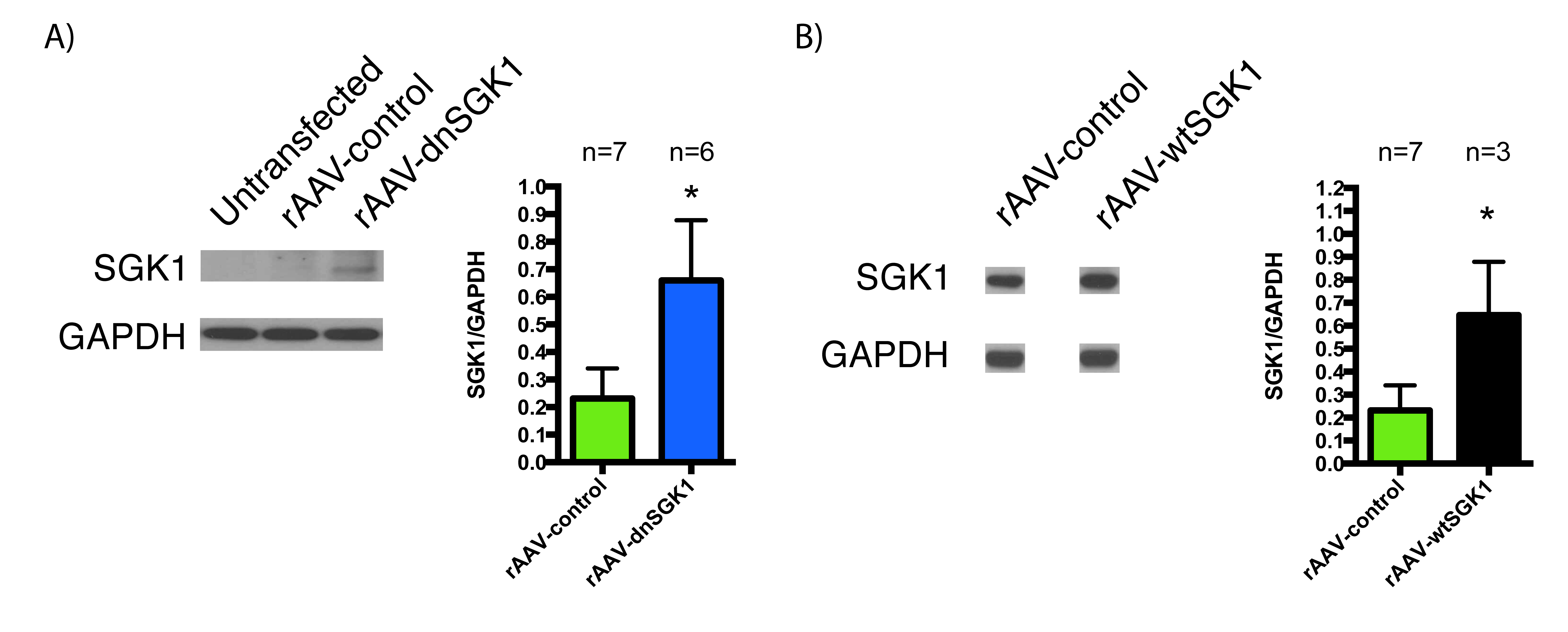

Supplement: S5 Fig — (TIF) [file pbio.1002282.s006.tif]

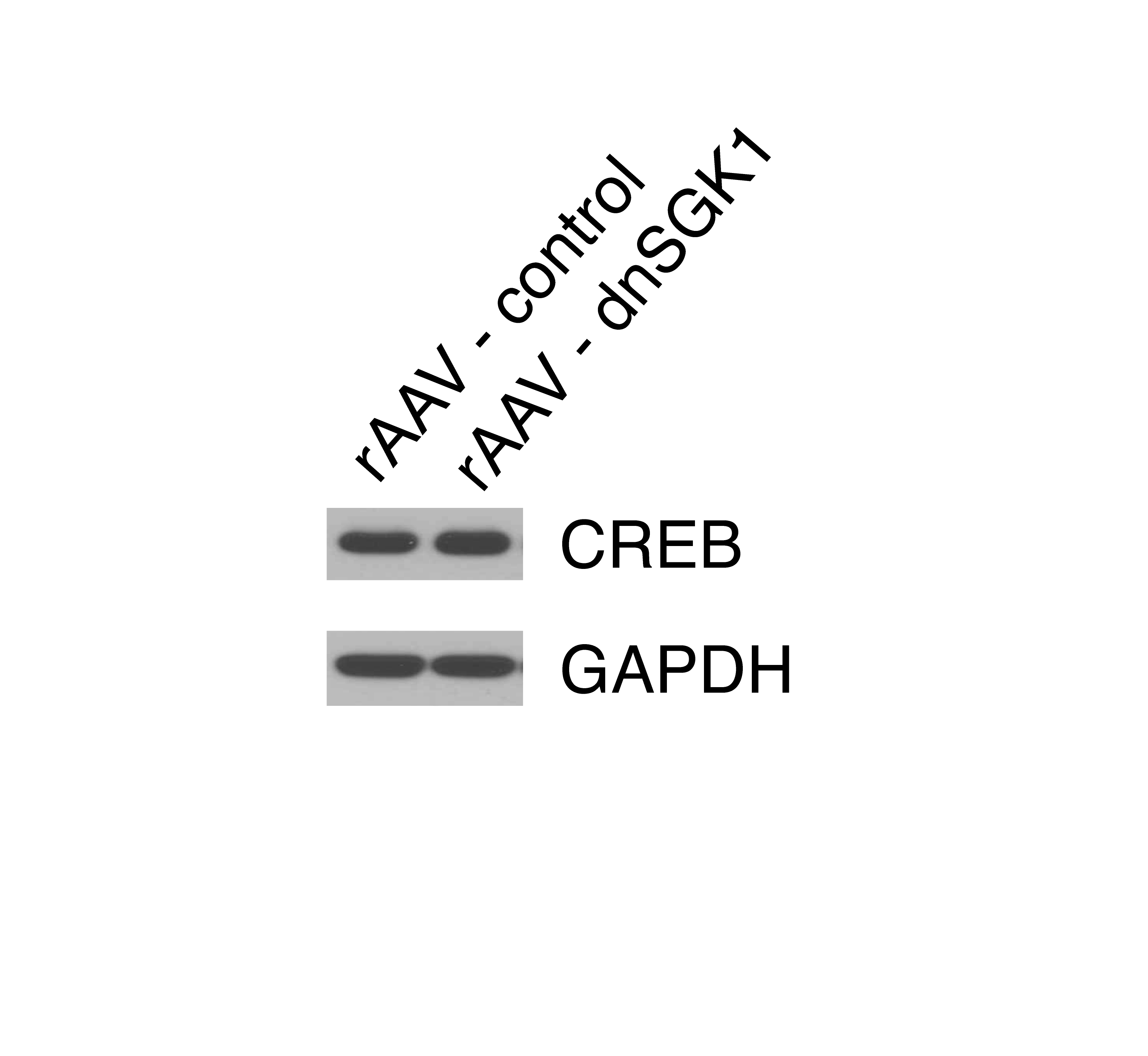

Supplement: S6 Fig — Experiment was replicated three times. (TIF) [file pbio.1002282.s007.tif]
