# Supplementary material for: Decreased SGK1 Expression and Function Contributes to Behavioral Deficits Induced by Traumatic Stress
Source: PLoS Biol. 2015 Oct 27;13(10):e1002282. doi: 10.1371/journal.pbio.1002282 (PMC4623974; doi:10.1371/journal.pbio.1002282)
Supplement: S1 Table — F, female; M, male; C, Caucasian; H, Hispanic; PMI, postmortem interval (hours); RIN, RNA integrity number. (DOCX) [file pbio.1002282.s008.docx]

| Sex/Race | Age | PMI | pH | RIN | Sex/Race | Age | PMI | pH | RIN |
| --- | --- | --- | --- | --- | --- | --- | --- | --- | --- |
| F/C | 38 | 41 | 6.76 | 8.0 | F/C | 42 | 36 | 6.58 | 7.7 |
| F/C | 39 | 33 | 6.1 | 8.3 | F/C | 40 | 38 | 6.35 | 8.7 |
| F/C | 34 | 54 | 6.21 | 5.7 | F/C | 39 | 39 | 6.45 | 6.5 |
| F/H | 56 | 54.5 | 6.4 | 7.8 | F/C | 47 | 46 | 6.77 | 8.7 |
| M/C | 53 | 63 | 6.17 | 8.3 | M/C | 54 | 86 | 6.77 | 8.2 |
| F | 45 | 25 | 6.74 | 7.6 | F | 44 | 46 | 6.56 | 8.2 |

**Control**

**PTSD**
