# Supplementary material for: Decreased SGK1 Expression and Function Contributes to Behavioral Deficits Induced by Traumatic Stress
Source: PLoS Biol. 2015 Oct 27;13(10):e1002282. doi: 10.1371/journal.pbio.1002282 (PMC4623974; doi:10.1371/journal.pbio.1002282)
Supplement: S2 Table — Complete list of up- and down-regulated genes after FDR adjustment for p-values. Healthy controls, n = 5; PTSD patients, n = 6. (DOCX) [file pbio.1002282.s009.docx]

| **Upregulated genes in the PFC of** | | |  |  |  |  |  |  |  |  |  |  |  |  |  |  |
| --- | --- | --- | --- | --- | --- | --- | --- | --- | --- | --- | --- | --- | --- | --- | --- | --- |
| **PTSD patients (> 1.5 Fold)** | | |  |  |  |  |  |  |  |  |  |  |  |  |  |  |
| pValue - FDR corrected probability | | |  |  |  |  |  |  |  |  |  |  |  |  |  |  |
|  |  |  |  |  |  |  |  |  |  |  |  |  |  |  |  |  |
|  |  |  |  |  |  |  |  |  |  |  |  |  |  |  |  |  |
|  |  |  |  |  |  |  |  |  |  |  |  |  |  |  |  |  |
|  |  |  |  |  |  |  |  |  |  |  |  |  |  |  |  |  |
|  |  |  |  |  |  |  |  |  |  |  |  |  |  |  |  |  |
| **Probe ID** | **p-value** | **Fold** |  | **Description** | |  |  |  |  |  |  |  |  |  |  |  |
| HOXA11 | 0.029 | 87.84543 |  | Homo sapiens homeo box A11 (HOXA11), mRNA. | | | | |  |  |  |  |  |  |  |  |
| FLJ36754 | 0.0259 | 28.583303 |  | - |  |  |  |  |  |  |  |  |  |  |  |  |
| USP40 | 0.0292 | 21.178617 |  | Homo sapiens ubiquitin specific protease 40 (USP40), mRNA. | | | | | |  |  |  |  |  |  |  |
| NKIRAS2 | 0.0183 | 21.158966 |  | Homo sapiens NFKB inhibitor interacting Ras-like 2 (NKIRAS2), transcript variant 2, mRNA. | | | | | | | |  |  |  |  |  |
| SURB7 | 0.015 | 9.382156 |  | Homo sapiens SRB7 suppressor of RNA polymerase B homolog (yeast) (SURB7), mRNA. | | | | | | | |  |  |  |  |  |
| KIN | 0.029 | 8.065217 |  | - |  |  |  |  |  |  |  |  |  |  |  |  |
| UTS2D | 0.0206 | 6.2957277 |  | - |  |  |  |  |  |  |  |  |  |  |  |  |
| UTS2D | 0.044648598 | 6.2957277 |  | - |  |  |  |  |  |  |  |  |  |  |  |  |
| TIMM50 | 0.0242 | 5.6496663 |  | Homo sapiens translocase of inner mitochondrial membrane 50 homolog (yeast) (TIMM50), mRNA. | | | | | | | |  |  |  |  |  |
| LOC200933 | 0.0146 | 5.2740364 |  | PREDICTED: Homo sapiens hypothetical protein LOC200933 (LOC200933), mRNA. | | | | | | | |  |  |  |  |  |
| POLD3 | 0.0146 | 4.943492 |  | Homo sapiens polymerase (DNA-directed), delta 3, accessory subunit (POLD3), mRNA. | | | | | | | |  |  |  |  |  |
| MSL3L1 | 0.0159 | 4.9126906 |  | Homo sapiens male-specific lethal 3-like 1 (Drosophila) (MSL3L1), transcript variant 3, mRNA. | | | | | | | |  |  |  |  |  |
| FLJ13798 | 0.029 | 4.627959 |  | Homo sapiens hypothetical protein FLJ13798 (FLJ13798), mRNA. | | | | | |  |  |  |  |  |  |  |
| LOC400304 | 0.029 | 4.401284 |  | PREDICTED: Homo sapiens similar to hypothetical protein (LOC400304), mRNA. | | | | | | |  |  |  |  |  |  |
| CACNA2D2 | 0.0146 | 3.8006697 |  | Homo sapiens calcium channel, voltage-dependent, alpha 2/delta subunit 2 (CACNA2D2), transcript variant 2, mRNA. | | | | | | | | | |  |  |  |
| BCL11B | 0.0223 | 3.723311 |  | Homo sapiens B-cell CLL/lymphoma 11B (zinc finger protein) (BCL11B), transcript variant 1, mRNA. | | | | | | | |  |  |  |  |  |
| BCL11B | 0.046074938 | 3.723311 |  | Homo sapiens B-cell CLL/lymphoma 11B (zinc finger protein) (BCL11B), transcript variant 1, mRNA. | | | | | | | |  |  |  |  |  |
| EIF4EBP3 | 0.0259 | 3.592938 |  | Homo sapiens eukaryotic translation initiation factor 4E binding protein 3 (EIF4EBP3), mRNA. | | | | | | | |  |  |  |  |  |
| FBXO16 | 0.029 | 3.3168027 |  | Homo sapiens F-box protein 16 (FBXO16), mRNA. | | | | |  |  |  |  |  |  |  |  |
| ZNF91 | 0.0146 | 3.215445 |  | Homo sapiens zinc finger protein 91 (HPF7, HTF10) (ZNF91), mRNA. | | | | | |  |  |  |  |  |  |  |
| LOC388348 | 0.0208 | 2.9370687 |  | PREDICTED: Homo sapiens hypothetical LOC388348 (LOC388348), mRNA. | | | | | | |  |  |  |  |  |  |
| LOC441283 | 0.0146 | 2.8052626 |  | PREDICTED: Homo sapiens hypothetical gene supported by AK131347 (LOC441283), mRNA. | | | | | | | |  |  |  |  |  |
| DLGAP4 | 4.96E-05 | 2.7991128 |  | Homo sapiens discs, large (Drosophila) homolog-associated protein 4 (DLGAP4), transcript variant 2, mRNA. | | | | | | | | |  |  |  |  |
| C1orf89 | 0.029 | 2.7948356 |  | - |  |  |  |  |  |  |  |  |  |  |  |  |
| HOM-TES-103 | 0.029 | 2.7419872 |  | Homo sapiens HOM-TES-103 tumor antigen-like (HOM-TES-103), transcript variant 3, mRNA. | | | | | | | |  |  |  |  |  |
| C1orf22 | 0.0146 | 2.6825266 |  | Homo sapiens chromosome 1 open reading frame 22 (C1orf22), mRNA. | | | | | | |  |  |  |  |  |  |
| MGC3329 | 0.0292 | 2.643564 |  | - |  |  |  |  |  |  |  |  |  |  |  |  |
| SSTR1 | 0.0259 | 2.639275 |  | Homo sapiens somatostatin receptor 1 (SSTR1), mRNA. | | | | |  |  |  |  |  |  |  |  |
| LSM10 | 0.0146 | 2.561252 |  | Homo sapiens LSM10, U7 small nuclear RNA associated (LSM10), mRNA. | | | | | | |  |  |  |  |  |  |
| LOC441848 | 0.00844 | 2.5395317 |  | PREDICTED: Homo sapiens similar to zinc finger protein 113 (LOC441848), mRNA. | | | | | | | |  |  |  |  |  |
| LOC393062 | 0.0259 | 2.532008 |  | PREDICTED: Homo sapiens similar to Mtr3 (mRNA transport regulator 3)-homolog; Mtr3 (mRNA transport regulator 3)-homolog (yeast) (LOC393062), mRNA. | | | | | | | | | | | | |
| LOC441742 | 0.029 | 2.4793148 |  | PREDICTED: Homo sapiens similar to Gamma-aminobutyric-acid receptor alpha-5 subunit precursor (GABA(A) receptor) (LOC441742), mRNA. | | | | | | | | | | | |  |
| XAB2 | 0.0146 | 2.3031154 |  | Homo sapiens XPA binding protein 2 (XAB2), mRNA. | | | | |  |  |  |  |  |  |  |  |
| PRKCABP | 0.0098 | 2.2105887 |  | Homo sapiens protein kinase C, alpha binding protein (PRKCABP), mRNA. | | | | | | |  |  |  |  |  |  |
| PRKCABP | 0.035377388 | 2.2105887 |  | Homo sapiens protein kinase C, alpha binding protein (PRKCABP), mRNA. | | | | | | |  |  |  |  |  |  |
| PRKCABP | 0.035377388 | 2.2105887 |  | Homo sapiens protein kinase C, alpha binding protein (PRKCABP), mRNA. | | | | | | |  |  |  |  |  |  |
| CLEC11A | 0.029 | 2.1082428 |  | Homo sapiens C-type lectin domain family 11, member A (CLEC11A), mRNA. | | | | | | |  |  |  |  |  |  |
| CACNA2D3 | 0.040369578 | 2.0921936 |  | Homo sapiens calcium channel, voltage-dependent, alpha 2/delta subunit 2 (CACNA2D2), transcript variant 2, mRNA. | | | | | | | | | |  |  |  |
| CACNA2D3 | 0.040369578 | 2.0921936 |  | Homo sapiens calcium channel, voltage-dependent, alpha 2/delta subunit 2 (CACNA2D2), transcript variant 2, mRNA. | | | | | | | | | |  |  |  |
| ASAH1 | 0.0292 | 1.7592579 |  | Homo sapiens N-acylsphingosine amidohydrolase (acid ceramidase) 1 (ASAH1), transcript variant 1, mRNA. | | | | | | | | |  |  |  |  |
| C1orf88 | 0.016084319 | 1.5550158 |  | Homo sapiens chromosome 1 open reading frame 87 (C1orf87), mRNA. | | | | | | |  |  |  |  |  |  |
| LOC441284 | 0.038943238 | 1.5070413 |  | PREDICTED: Homo sapiens hypothetical gene supported by AK131347 (LOC441283), mRNA. | | | | | | | |  |  |  |  |  |
| LOC441284 | 0.038943238 | 1.5070413 |  | PREDICTED: Homo sapiens hypothetical gene supported by AK131347 (LOC441283), mRNA. | | | | | | | |  |  |  |  |  |
|  |  |  |  |  |  |  |  |  |  |  |  |  |  |  |  |  |
|  |  |  |  |  |  |  |  |  |  |  |  |  |  |  |  |  |

| **Downregulated genes in the PFC of** | | | |  |  |  |  |  |  |  |  |  |  |  |
| --- | --- | --- | --- | --- | --- | --- | --- | --- | --- | --- | --- | --- | --- | --- |
| **PTSD patients (> 1.5 Fold)** | | |  |  |  |  |  |  |  |  |  |  |  |  |
| pValue - FDR corrected probability | | |  |  |  |  |  |  |  |  |  |  |  |  |
|  |  |  |  |  |  |  |  |  |  |  |  |  |  |  |
|  |  |  |  |  |  |  |  |  |  |  |  |  |  |  |
|  |  |  |  |  |  |  |  |  |  |  |  |  |  |  |
|  |  |  |  |  |  |  |  |  |  |  |  |  |  |  |
|  |  |  |  |  |  |  |  |  |  |  |  |  |  |  |
| **Probe ID** | **p-value** | **Fold** |  | **Description** | |  |  |  |  |  |  |  |  |  |
| LOC342994 | 0.0465 | 0.7058837 |  | PREDICTED: Homo sapiens similar to ribosomal protein L34; 60S ribosomal protein L34 (LOC342994), mRNA. | | | | | | | | | |  |
| POLD4 | 0.036090558 | 0.69803953 |  | Homo sapiens polymerase (DNA-directed), delta 4 (POLD4), mRNA. | | | | | |  |  |  |  |  |
| ARHGEF7 | 0.0471 | 0.67150056 |  | Homo sapiens Rho guanine nucleotide exchange factor (GEF) 7 (ARHGEF7), transcript variant 1, mRNA. | | | | | | | | |  |  |
| LOC441849 | 0.034664218 | 0.6640071 |  | PREDICTED: Homo sapiens similar to Methionine-R-sulfoxide reductase (Selenoprotein X 1) (HSPC270) (LOC441849), mRNA. | | | | | | | | | | |
| MMD | 0.029 | 0.6637945 |  | Homo sapiens monocyte to macrophage differentiation-associated (MMD), mRNA. | | | | | | | |  |  |  |
| ZNF92 | 0.038230068 | 0.6570474 |  | Homo sapiens zinc finger protein 92 (HTF12) (ZNF92), mRNA. | | | | | |  |  |  |  |  |
| PHLDA3 | 0.042 | 0.6532847 |  | - |  |  |  |  |  |  |  |  |  |  |
| LOC388885 | 0.042 | 0.65297866 |  | PREDICTED: Homo sapiens hypothetical LOC388885 (LOC388885), mRNA. | | | | | | |  |  |  |  |
| LANCL2 | 0.029 | 0.6447031 |  | Homo sapiens LanC lantibiotic synthetase component C-like 2 (bacterial) (LANCL2), mRNA. | | | | | | | |  |  |  |
| LYPLA2P1 | 0.0434 | 0.63949317 |  | - |  |  |  |  |  |  |  |  |  |  |
| MAP7 | 0.0387 | 0.6364066 |  | Homo sapiens microtubule-associated protein 7 (MAP7), mRNA. | | | | | |  |  |  |  |  |
| C9orf97 | 0.0471 | 0.63361347 |  | Homo sapiens chromosome 9 open reading frame 97 (C9orf97), mRNA. | | | | | | |  |  |  |  |
| LOC341511 | 0.0497 | 0.62502795 |  | PREDICTED: Homo sapiens similar to 60S ribosomal protein L23a (LOC341511), mRNA. | | | | | | | |  |  |  |
| FEZ1 | 0.0473 | 0.6049123 |  | Homo sapiens fasciculation and elongation protein zeta 1 (zygin I) (FEZ1), transcript variant 1, mRNA. | | | | | | | | |  |  |
| LOC440575 | 0.0452 | 0.6003433 |  | PREDICTED: Homo sapiens similar to ribosomal protein L21 (LOC440575), mRNA. | | | | | | | |  |  |  |
| SURF4 | 0.029 | 0.59688044 |  | Homo sapiens surfeit 4 (SURF4), mRNA. | | | |  |  |  |  |  |  |  |
| BMX | 0.0471 | 0.54305595 |  | Homo sapiens BMX non-receptor tyrosine kinase (BMX), mRNA. | | | | | |  |  |  |  |  |
| CPSF3 | 0.0497 | 0.53760743 |  | Homo sapiens cleavage and polyadenylation specific factor 3, 73kDa (CPSF3), mRNA. | | | | | | | |  |  |  |
| SOX2OT | 0.0448 | 0.53423697 |  | - |  |  |  |  |  |  |  |  |  |  |
| MASA | 0.0408 | 0.53394884 |  | Homo sapiens E-1 enzyme (MASA), mRNA. | | | |  |  |  |  |  |  |  |
| CNTNAP4 | 0.0055 | 0.5242611 |  | - |  |  |  |  |  |  |  |  |  |  |
| PTP4A2 | 0.0408 | 0.5226999 |  | Homo sapiens protein tyrosine phosphatase type IVA, member 2 (PTP4A2), transcript variant 1, mRNA. | | | | | | | | |  |  |
| DNAH1 | 0.0452 | 0.51171243 |  | Homo sapiens dynein, axonemal, heavy polypeptide 1 (DNAH1), mRNA. | | | | | | |  |  |  |  |
| SERPINA12 | 0.042 | 0.5102148 |  | Homo sapiens serine (or cysteine) proteinase inhibitor, clade A (alpha-1 antiproteinase, antitrypsin), member 12 (SERPINA12), mRNA. | | | | | | | | | | |
| FTHL11 | 0.0434 | 0.5053909 |  | Homo sapiens ferritin, heavy polypeptide-like 11 (FTHL11) on chromosome 8. | | | | | | |  |  |  |  |
| GAB3 | 0.01401805 | 0.5034521 |  | Homo sapiens GRB2-associated binding protein 2 (GAB2), transcript variant 2, mRNA. | | | | | | | |  |  |  |
| RNF138 | 0.00809 | 0.49576306 |  | - |  |  |  |  |  |  |  |  |  |  |
| LOC387921 | 0.0374 | 0.49269587 |  | - |  |  |  |  |  |  |  |  |  |  |
| H2AFJ | 0.029 | 0.48314822 |  | Homo sapiens H2A histone family, member J (H2AFJ), transcript variant 1, mRNA. | | | | | | | |  |  |  |
| C6orf134 | 0.0448 | 0.483061 |  | - |  |  |  |  |  |  |  |  |  |  |
| MGC10067 | 0.0288 | 0.48272514 |  | Homo sapiens ubiquitin-like domain containing CTD phosphatase 1 (MGC10067), mRNA. | | | | | | | |  |  |  |
| PPAP2A | 0.0384 | 0.48195958 |  | Homo sapiens phosphatidic acid phosphatase type 2A (PPAP2A), transcript variant 1, mRNA. | | | | | | | | |  |  |
| PICALM | 0.0387 | 0.477162 |  | Homo sapiens phosphatidylinositol binding clathrin assembly protein (PICALM), transcript variant 1, mRNA. | | | | | | | | | |  |
| LISCH7 | 0.0101 | 0.46428427 |  | Homo sapiens liver-specific bHLH-Zip transcription factor (LISCH7), transcript variant 1, mRNA. | | | | | | | | |  |  |
| PAPPA2 | 0.0387 | 0.45652905 |  | Homo sapiens pappalysin 2 (PAPPA2), transcript variant 1, mRNA. | | | | | |  |  |  |  |  |
| UBE2J1 | 0.0288 | 0.45546716 |  | Homo sapiens ubiquitin-conjugating enzyme E2, J1 (UBC6 homolog, yeast) (UBE2J1), mRNA. | | | | | | | |  |  |  |
| EPS8L1 | 0.0384 | 0.4519577 |  | Homo sapiens EPS8-like 1 (EPS8L1), transcript variant 3, mRNA. | | | | | |  |  |  |  |  |
| GOLGA7 | 0.0387 | 0.4506875 |  | Homo sapiens golgi autoantigen, golgin subfamily a, 7 (GOLGA7), transcript variant 1, mRNA. | | | | | | | | |  |  |
| CSMD1 | 0.0374 | 0.44870988 |  | Homo sapiens CUB and Sushi multiple domains 1 (CSMD1), mRNA. | | | | | |  |  |  |  |  |
| POU3F4 | 0.0479 | 0.44619164 |  | Homo sapiens POU domain, class 3, transcription factor 4 (POU3F4), mRNA. | | | | | | |  |  |  |  |
| SASH1 | 0.0492 | 0.44479647 |  | Homo sapiens SAM and SH3 domain containing 1 (SASH1), mRNA. | | | | | |  |  |  |  |  |
| HTLF | 0.0288 | 0.44335902 |  | Homo sapiens human T-cell leukemia virus enhancer factor (HTLF), mRNA. | | | | | | |  |  |  |  |
| FLII | 0.0255 | 0.43974707 |  | Homo sapiens flightless I homolog (Drosophila) (FLII), mRNA. | | | | | |  |  |  |  |  |
| JAM3 | 0.0408 | 0.43387774 |  | Homo sapiens junctional adhesion molecule 3 (JAM3), mRNA. | | | | | |  |  |  |  |  |
| NDRG1 | 0.0276 | 0.43191645 |  | Homo sapiens N-myc downstream regulated gene 1 (NDRG1), mRNA. | | | | | |  |  |  |  |  |
| DKFZP434A0131 | 0.044 | 0.42887145 |  | Homo sapiens DKFZp434A0131 protein (DKFZP434A0131), transcript variant 1, mRNA. | | | | | | | |  |  |  |
| KIAA0103 | 0.0471 | 0.42568362 |  | - |  |  |  |  |  |  |  |  |  |  |
| RSNL2 | 0.028 | 0.42555544 |  | Homo sapiens restin-like 2 (RSNL2), mRNA. | | | |  |  |  |  |  |  |  |
| CSPG2 | 0.00491 | 0.42390695 |  | Homo sapiens chondroitin sulfate proteoglycan 2 (versican) (CSPG2), mRNA. | | | | | | |  |  |  |  |
| C14orf46 | 0.00809 | 0.42309356 |  | Homo sapiens chromosome 14 open reading frame 46 (C14orf46), mRNA. | | | | | | |  |  |  |  |
| ABCA2 | 0.0497 | 0.41902906 |  | Homo sapiens ATP-binding cassette, sub-family A (ABC1), member 2 (ABCA2), transcript variant 2, mRNA. | | | | | | | | | |  |
| CDK5RAP2 | 0.0452 | 0.4113454 |  | Homo sapiens CDK5 regulatory subunit associated protein 2 (CDK5RAP2), transcript variant 2, mRNA. | | | | | | | | |  |  |
| C10orf56 | 0.029 | 0.40733093 |  | Homo sapiens chromosome 10 open reading frame 56 (C10orf56), mRNA. | | | | | | |  |  |  |  |
| TAF1A | 0.0126 | 0.4061119 |  | Homo sapiens TATA box binding protein (TBP)-associated factor, RNA polymerase I, A, 48kDa (TAF1A), transcript variant 1, mRNA. | | | | | | | | | | |
| CDC42SE1 | 0.0408 | 0.40456894 |  | Homo sapiens CDC42 small effector 1 (CDC42SE1), mRNA. | | | | | |  |  |  |  |  |
| NOTCH4 | 0.0497 | 0.40046996 |  | Homo sapiens Notch homolog 4 (Drosophila) (NOTCH4), mRNA. | | | | | |  |  |  |  |  |
| ANKRD27 | 0.0288 | 0.3981657 |  | Homo sapiens ankyrin repeat domain 27 (VPS9 domain) (ANKRD27), mRNA. | | | | | | |  |  |  |  |
| HADHB | 0.0311 | 0.3956527 |  | Homo sapiens hydroxyacyl-Coenzyme A dehydrogenase/3-ketoacyl-Coenzyme A thiolase/enoyl-Coenzyme A hydratase (trifunctional protein), beta subunit (HADHB), mRNA. | | | | | | | | | | |
| NR4A3 | 0.0101 | 0.39562315 |  | Homo sapiens nuclear receptor subfamily 4, group A, member 3 (NR4A3), transcript variant 2, mRNA. | | | | | | | | |  |  |
| NEDD9 | 0.044 | 0.39053237 |  | Homo sapiens neural precursor cell expressed, developmentally down-regulated 9 (NEDD9), mRNA. | | | | | | | | |  |  |
| CNGA2 | 0.0492 | 0.38784394 |  | Homo sapiens cyclic nucleotide gated channel alpha 2 (CNGA2), mRNA. | | | | | | |  |  |  |  |
| ST18 | 0.0479 | 0.38392946 |  | Homo sapiens suppression of tumorigenicity 18 (breast carcinoma) (zinc finger protein) (ST18), mRNA. | | | | | | | | |  |  |
| FBXO9 | 0.0497 | 0.38343722 |  | Homo sapiens F-box protein 9 (FBXO9), transcript variant 2, mRNA. | | | | | |  |  |  |  |  |
| GAB2 | 0.0055 | 0.38189867 |  | Homo sapiens GRB2-associated binding protein 2 (GAB2), transcript variant 2, mRNA. | | | | | | | |  |  |  |
| FLJ90119 | 0.0471 | 0.38145787 |  | Homo sapiens hypothetical protein FLJ90119 (FLJ90119), mRNA. | | | | | |  |  |  |  |  |
| PCBP4 | 0.00491 | 0.381213 |  | Homo sapiens poly(rC) binding protein 4 (PCBP4), transcript variant 4, mRNA. | | | | | | |  |  |  |  |
| RARSL | 0.0485 | 0.38077152 |  | Homo sapiens arginyl-tRNA synthetase-like (RARSL), mRNA. | | | | | |  |  |  |  |  |
| NKX2-2 | 0.0303 | 0.38044015 |  | Homo sapiens NK2 transcription factor related, locus 2 (Drosophila) (NKX2-2), mRNA. | | | | | | | |  |  |  |
| GAL3ST1 | 0.0452 | 0.37999082 |  | Homo sapiens galactose-3-O-sulfotransferase 1 (GAL3ST1), mRNA. | | | | | |  |  |  |  |  |
| COX11 | 0.029 | 0.37836123 |  | - |  |  |  |  |  |  |  |  |  |  |
| LZTS2 | 0.042 | 0.3746457 |  | Homo sapiens leucine zipper, putative tumor suppressor 2 (LZTS2), mRNA. | | | | | | |  |  |  |  |
| RAB8A | 0.0285 | 0.3739841 |  | Homo sapiens RAB8A, member RAS oncogene family (RAB8A), mRNA. | | | | | |  |  |  |  |  |
| RASAL1 | 0.0492 | 0.3723949 |  | - |  |  |  |  |  |  |  |  |  |  |
| SCD | 0.0492 | 0.37085178 |  | Homo sapiens stearoyl-CoA desaturase (delta-9-desaturase) (SCD), mRNA. | | | | | | |  |  |  |  |
| GATM | 0.0319 | 0.3682254 |  | - |  |  |  |  |  |  |  |  |  |  |
| LOC150928 | 0.0322 | 0.3680386 |  | - |  |  |  |  |  |  |  |  |  |  |
| ARHGEF10 | 0.044 | 0.3632815 |  | Homo sapiens Rho guanine nucleotide exchange factor (GEF) 10 (ARHGEF10), mRNA. | | | | | | | |  |  |  |
| DTX2 | 0.0497 | 0.3627916 |  | Homo sapiens deltex homolog 2 (Drosophila) (DTX2), mRNA. | | | | | |  |  |  |  |  |
| EGR2 | 0.0444 | 0.35630548 |  | - |  |  |  |  |  |  |  |  |  |  |
| EGFL9 | 0.00491 | 0.35203797 |  | Homo sapiens EGF-like-domain, multiple 9 (EGFL9), transcript variant 1, mRNA. | | | | | | |  |  |  |  |
| EDG2 | 0.00809 | 0.35100892 |  | Homo sapiens endothelial differentiation, lysophosphatidic acid G-protein-coupled receptor, 2 (EDG2), transcript variant 1, mRNA. | | | | | | | | | | |
| C14orf32 | 0.042 | 0.34424508 |  | Homo sapiens chromosome 14 open reading frame 32 (C14orf32), mRNA. | | | | | | |  |  |  |  |
| HEY2 | 0.0288 | 0.34409708 |  | Homo sapiens hairy/enhancer-of-split related with YRPW motif 2 (HEY2), mRNA. | | | | | | |  |  |  |  |
| TM4SF11 | 0.0306 | 0.33255133 |  | Homo sapiens transmembrane 4 superfamily member 11 (plasmolipin) (TM4SF11), mRNA. | | | | | | | |  |  |  |
| SETDB2 | 0.029 | 0.32966122 |  | Homo sapiens SET domain, bifurcated 2 (SETDB2), mRNA. | | | | |  |  |  |  |  |  |
| NEK11 | 0.00809 | 0.32728168 |  | Homo sapiens NIMA (never in mitosis gene a)- related kinase 11 (NEK11), mRNA. | | | | | | |  |  |  |  |
| FXYD4 | 0.0279 | 0.3267725 |  | Homo sapiens FXYD domain containing ion transport regulator 4 (FXYD4), mRNA. | | | | | | | |  |  |  |
| WDR8 | 0.0497 | 0.32612702 |  | Homo sapiens WD repeat domain 8 (WDR8), mRNA. | | | | |  |  |  |  |  |  |
| C1orf108 | 0.0303 | 0.31702062 |  | - |  |  |  |  |  |  |  |  |  |  |
| LOC389827 | 0.0497 | 0.31562492 |  | PREDICTED: Homo sapiens similar to RIKEN cDNA 1110002H13 (LOC389827), mRNA. | | | | | | | |  |  |  |
| SYNGR2 | 0.0355 | 0.31465286 |  | Homo sapiens synaptogyrin 2 (SYNGR2), mRNA. | | | | |  |  |  |  |  |  |
| GLI3 | 0.0455 | 0.31343365 |  | Homo sapiens GLI-Kruppel family member GLI3 (Greig cephalopolysyndactyly syndrome) (GLI3), mRNA. | | | | | | | | |  |  |
| ALMS1 | 0.0434 | 0.31126907 |  | Homo sapiens Alstrom syndrome 1 (ALMS1), mRNA. | | | | |  |  |  |  |  |  |
| FLJ14525 | 0.0452 | 0.30747968 |  | Homo sapiens hypothetical protein FLJ14525 (FLJ14525), mRNA. | | | | | |  |  |  |  |  |
| FNBP4 | 0.0384 | 0.30326125 |  | Homo sapiens formin binding protein 4 (FNBP4), mRNA. | | | | |  |  |  |  |  |  |
| LOC150356 | 0.0387 | 0.2959113 |  | - |  |  |  |  |  |  |  |  |  |  |
| DUSP10 | 0.00809 | 0.2897017 |  | Homo sapiens dual specificity phosphatase 10 (DUSP10), transcript variant 3, mRNA. | | | | | | | |  |  |  |
| BOLA3 | 0.0055 | 0.2890992 |  | Homo sapiens bolA-like 3 (E. coli) (BOLA3), mRNA. | | | | |  |  |  |  |  |  |
| HNRPLL | 0.0444 | 0.28690517 |  | Homo sapiens heterogeneous nuclear ribonucleoprotein L-like (HNRPLL), mRNA. | | | | | | | |  |  |  |
| LIPT1 | 0.0288 | 0.2864731 |  | Homo sapiens lipoyltransferase 1 (LIPT1), transcript variant 3, mRNA. | | | | | | |  |  |  |  |
| wdr16 | 0.0408 | 0.28640842 |  | Homo sapiens hypothetical protein LOC146845 (wdr16), mRNA. | | | | | |  |  |  |  |  |
| C14orf24 | 0.0306 | 0.28610817 |  | Homo sapiens chromosome 14 open reading frame 24 (C14orf24), mRNA. | | | | | | |  |  |  |  |
| LAMP2 | 0.00293 | 0.28421035 |  | Homo sapiens lysosomal-associated membrane protein 2 (LAMP2), transcript variant LAMP2B, mRNA. | | | | | | | | |  |  |
| CXCL2 | 0.042 | 0.28337204 |  | - |  |  |  |  |  |  |  |  |  |  |
| LOC441323 | 0.0497 | 0.2796535 |  | PREDICTED: Homo sapiens similar to FLJ10408 protein (LOC441323), mRNA. | | | | | | |  |  |  |  |
| ZNF77 | 0.0452 | 0.27360147 |  | Homo sapiens zinc finger protein 77 (pT1) (ZNF77), mRNA. | | | | | |  |  |  |  |  |
| STXBP3 | 0.0288 | 0.27166277 |  | Homo sapiens syntaxin binding protein 3 (STXBP3), mRNA. | | | | | |  |  |  |  |  |
| RDH10 | 0.0288 | 0.27162147 |  | Homo sapiens retinol dehydrogenase 10 (all-trans) (RDH10), mRNA. | | | | | |  |  |  |  |  |
| MYLK | 0.0101 | 0.27148214 |  | Homo sapiens myosin, light polypeptide kinase (MYLK), transcript variant 5, mRNA. | | | | | | | |  |  |  |
| CLMN | 0.0374 | 0.26993364 |  | Homo sapiens calmin (calponin-like, transmembrane) (CLMN), mRNA. | | | | | | |  |  |  |  |
| TMPRSS11A | 0.0434 | 0.26980868 |  | Homo sapiens transmembrane protease, serine 11A (TMPRSS11A), mRNA. | | | | | | |  |  |  |  |
| C18orf43 | 0.0387 | 0.2682904 |  | Homo sapiens chromosome 18 open reading frame 43 (C18orf43), mRNA. | | | | | | |  |  |  |  |
| ZBED3 | 0.0448 | 0.26667967 |  | Homo sapiens zinc finger, BED-type containing 3 (ZBED3), mRNA. | | | | | |  |  |  |  |  |
| CNKSR3 | 0.044 | 0.26606822 |  | Homo sapiens CNKSR family member 3 (CNKSR3), mRNA. | | | | |  |  |  |  |  |  |
| SEMA3B | 0.0485 | 0.2644909 |  | Homo sapiens sema domain, immunoglobulin domain (Ig), short basic domain, secreted, (semaphorin) 3B (SEMA3B), transcript variant 1, mRNA. | | | | | | | | | | |
| KIAA1189 | 0.0448 | 0.26189223 |  | Homo sapiens KIAA1189 (KIAA1189), transcript variant 2, mRNA. | | | | | |  |  |  |  |  |
| PPAT | 0.0306 | 0.25515667 |  | Homo sapiens phosphoribosyl pyrophosphate amidotransferase (PPAT), mRNA. | | | | | | |  |  |  |  |
| RAB41 | 0.0387 | 0.254088 |  | PREDICTED: Homo sapiens RAB41, member RAS homolog family (RAB41), mRNA. | | | | | | |  |  |  |  |
| SLC15A2 | 0.0381 | 0.25004828 |  | Homo sapiens solute carrier family 15 (H+/peptide transporter), member 2 (SLC15A2), mRNA. | | | | | | | | |  |  |
| RNF103 | 0.0471 | 0.2493747 |  | Homo sapiens ring finger protein 103 (RNF103), mRNA. | | | | |  |  |  |  |  |  |
| ALDH9A1 | 0.0485 | 0.24930258 |  | Homo sapiens aldehyde dehydrogenase 9 family, member A1 (ALDH9A1), mRNA. | | | | | | |  |  |  |  |
| FLJ22635 | 0.0485 | 0.24445963 |  | Homo sapiens hypothetical protein FLJ22635 (FLJ22635), mRNA. | | | | | |  |  |  |  |  |
| LOC256176 | 0.0448 | 0.24111074 |  | PREDICTED: Homo sapiens hypothetical LOC256176 (LOC256176), mRNA. | | | | | | |  |  |  |  |
| LOC349236 | 0.0332 | 0.24069855 |  | Homo sapiens hypothetical protein LOC349236 (LOC349236), mRNA. | | | | | | |  |  |  |  |
| GUCA2A | 0.0303 | 0.238845 |  | Homo sapiens guanylate cyclase activator 2A (guanylin) (GUCA2A), mRNA. | | | | | | |  |  |  |  |
| KIAA1946 | 0.0471 | 0.2350175 |  | Homo sapiens KIAA1946 (KIAA1946), mRNA. | | | |  |  |  |  |  |  |  |
| CSNK2A1P | 0.0126 | 0.2279328 |  | Homo sapiens casein kinase 2, alpha 1 polypeptide pseudogene (CSNK2A1P) on chromosome 11. | | | | | | | | |  |  |
| EXTL2 | 0.0387 | 0.22742796 |  | Homo sapiens exostoses (multiple)-like 2 (EXTL2), mRNA. | | | | |  |  |  |  |  |  |
| KIAA1666 | 0.0479 | 0.22721767 |  | PREDICTED: Homo sapiens KIAA1666 protein (KIAA1666), mRNA. | | | | | |  |  |  |  |  |
| IFNA6 | 0.0434 | 0.22518228 |  | Homo sapiens interferon, alpha 6 (IFNA6), mRNA. | | | | |  |  |  |  |  |  |
| KRTAP13-3 | 0.0387 | 0.22444421 |  | Homo sapiens keratin associated protein 13-3 (KRTAP13-3), mRNA. | | | | | |  |  |  |  |  |
| DDB2 | 0.00809 | 0.22267617 |  | Homo sapiens damage-specific DNA binding protein 2, 48kDa (DDB2), mRNA. | | | | | | |  |  |  |  |
| UPF3B | 0.017232246 | 0.22170846 |  | - |  |  |  |  |  |  |  |  |  |  |
| FA2H | 0.0357 | 0.22065613 |  | Homo sapiens fatty acid 2-hydroxylase (FA2H), mRNA. | | | | |  |  |  |  |  |  |
| ASXL1 | 0.0101 | 0.21878877 |  | Homo sapiens additional sex combs like 1 (Drosophila) (ASXL1), mRNA. | | | | | | |  |  |  |  |
| KRT6IRS | 0.0101 | 0.21850325 |  | Homo sapiens keratin 6 irs (KRT6IRS), mRNA. | | | |  |  |  |  |  |  |  |
| RALGDS | 0.0259 | 0.21525417 |  | - |  |  |  |  |  |  |  |  |  |  |
| KIAA1394 | 0.0452 | 0.20900965 |  | PREDICTED: Homo sapiens KIAA1394 protein (KIAA1394), mRNA. | | | | | |  |  |  |  |  |
| FLJ30707 | 0.0387 | 0.20896028 |  | - |  |  |  |  |  |  |  |  |  |  |
| USP51 | 0.0497 | 0.20785144 |  | Homo sapiens ubiquitin specific protease 51 (USP51), mRNA. | | | | | |  |  |  |  |  |
| LOC154761 | 0.0448 | 0.2075732 |  | - |  |  |  |  |  |  |  |  |  |  |
| PI16 | 0.0497 | 0.20679003 |  | Homo sapiens protease inhibitor 16 (PI16), mRNA. | | | | |  |  |  |  |  |  |
| LOC440585 | 0.0471 | 0.20602784 |  | PREDICTED: Homo sapiens LOC440585 (LOC440585), mRNA. | | | | | |  |  |  |  |  |
| FLJ30277 | 0.044 | 0.20591335 |  | Homo sapiens hypothetical protein FLJ30277 (FLJ30277), mRNA. | | | | | |  |  |  |  |  |
| CKLFSF2 | 0.0492 | 0.20565692 |  | Homo sapiens chemokine-like factor superfamily 2 (CKLFSF2), mRNA. | | | | | | |  |  |  |  |
| NUP88 | 0.029 | 0.20444307 |  | Homo sapiens nucleoporin 88kDa (NUP88), mRNA. | | | | |  |  |  |  |  |  |
| BARD1 | 0.00809 | 0.20301467 |  | Homo sapiens BRCA1 associated RING domain 1 (BARD1), mRNA. | | | | | |  |  |  |  |  |
| LOC199964 | 0.0303 | 0.20185177 |  | - |  |  |  |  |  |  |  |  |  |  |
| LOC196541 | 0.0128 | 0.20134322 |  | Homo sapiens OTTHUMP00000018663 (LOC196541), mRNA. | | | | | |  |  |  |  |  |
| PRAME | 0.00991 | 0.20084585 |  | Homo sapiens preferentially expressed antigen in melanoma (PRAME), transcript variant 1, mRNA. | | | | | | | | |  |  |
| NIPA1 | 0.00809 | 0.19979711 |  | Homo sapiens non imprinted in Prader-Willi/Angelman syndrome 1 (NIPA1), mRNA. | | | | | | | |  |  |  |
| C2orf15 | 0.0434 | 0.19899616 |  | Homo sapiens chromosome 2 open reading frame 15 (C2orf15), mRNA. | | | | | | |  |  |  |  |
| MME | 0.029 | 0.1977147 |  | Homo sapiens membrane metallo-endopeptidase (neutral endopeptidase, enkephalinase, CALLA, CD10) (MME), transcript variant 1bis, mRNA. | | | | | | | | | | |
| DYSF | 0.044 | 0.19320762 |  | Homo sapiens dysferlin, limb girdle muscular dystrophy 2B (autosomal recessive) (DYSF), mRNA. | | | | | | | | |  |  |
| NID67 | 0.0387 | 0.1920926 |  | Homo sapiens putative small membrane protein NID67 (NID67), mRNA. | | | | | | |  |  |  |  |
| LOC391428 | 0.042 | 0.19061646 |  | PREDICTED: Homo sapiens similar to Selenide,water dikinase 1 (Selenophosphate synthetase 1) (Selenium donor protein 1) (LOC391428), mRNA. | | | | | | | | | | |
| FANCD2 | 0.0492 | 0.1883839 |  | Homo sapiens Fanconi anemia, complementation group D2 (FANCD2), transcript variant 2, mRNA. | | | | | | | | |  |  |
| ELF5 | 0.00809 | 0.1866781 |  | Homo sapiens E74-like factor 5 (ets domain transcription factor) (ELF5), transcript variant 2, mRNA. | | | | | | | | |  |  |
| POLN | 0.0497 | 0.18587108 |  | Homo sapiens polymerase (DNA directed) nu (POLN), mRNA. | | | | | |  |  |  |  |  |
| HRASLS3 | 0.0497 | 0.18400227 |  | - |  |  |  |  |  |  |  |  |  |  |
| ZNF490 | 0.0404 | 0.18253927 |  | Homo sapiens zinc finger protein 490 (ZNF490), mRNA. | | | | |  |  |  |  |  |  |
| FLJ38991 | 0.0434 | 0.18116298 |  | Homo sapiens hypothetical protein FLJ38991 (FLJ38991), mRNA. | | | | | |  |  |  |  |  |
| FLJ33069 | 0.0288 | 0.17618304 |  | Homo sapiens hypothetical protein FLJ33069 (FLJ33069), mRNA. | | | | | |  |  |  |  |  |
| NALP8 | 0.029 | 0.17368986 |  | Homo sapiens NACHT, leucine rich repeat and PYD containing 8 (NALP8), mRNA. | | | | | | |  |  |  |  |
| MYCT1 | 0.0493 | 0.16799235 |  | Homo sapiens myc target 1 (MYCT1), mRNA. | | | |  |  |  |  |  |  |  |
| SLC5A11 | 0.0387 | 0.16551137 |  | Homo sapiens solute carrier family 5 (sodium/glucose cotransporter), member 11 (SLC5A11), mRNA. | | | | | | | | |  |  |
| VANGL2 | 0.0497 | 0.16522236 |  | Homo sapiens vang-like 2 (van gogh, Drosophila) (VANGL2), mRNA. | | | | | |  |  |  |  |  |
| TMEM10 | 0.0306 | 0.16512644 |  | Homo sapiens transmembrane protein 10 (TMEM10), mRNA. | | | | | |  |  |  |  |  |
| MGC17299 | 0.0101 | 0.16404563 |  | Homo sapiens hypothetical protein MGC17299 (MGC17299), mRNA. | | | | | | |  |  |  |  |
| LOC440935 | 0.0485 | 0.16269268 |  | PREDICTED: Homo sapiens LOC440935 (LOC440935), mRNA. | | | | | |  |  |  |  |  |
| SCAMP1 | 0.0492 | 0.16222529 |  | Homo sapiens secretory carrier membrane protein 1 (SCAMP1), transcript variant 2, mRNA. | | | | | | | |  |  |  |
| LOC441937 | 0.0306 | 0.16200475 |  | PREDICTED: Homo sapiens similar to hypothetical protein (LOC441937), mRNA. | | | | | | |  |  |  |  |
| CPEB3 | 0.0497 | 0.16094063 |  | Homo sapiens cytoplasmic polyadenylation element binding protein 3 (CPEB3), mRNA. | | | | | | | |  |  |  |
| TPST1 | 0.0128 | 0.15940921 |  | Homo sapiens tyrosylprotein sulfotransferase 1 (TPST1), mRNA. | | | | | |  |  |  |  |  |
| SGK | 0.0434 | 0.15892859 |  | Homo sapiens serum/glucocorticoid regulated kinase (SGK), mRNA. | | | | | |  |  |  |  |  |
| MOBKL1A | 0.0101 | 0.15787065 |  | Homo sapiens MOB1, Mps One Binder kinase activator-like 1A (yeast) (MOBKL1A), mRNA. | | | | | | | |  |  |  |
| LOC390243 | 0.044 | 0.15783896 |  | PREDICTED: Homo sapiens similar to folate receptor 3 (LOC390243), mRNA. | | | | | | |  |  |  |  |
| LOC441313 | 0.044 | 0.15705352 |  | PREDICTED: Homo sapiens similar to FLJ10408 protein (LOC441313), mRNA. | | | | | | |  |  |  |  |
| SMARCE1 | 0.029 | 0.15615924 |  | Homo sapiens SWI/SNF related, matrix associated, actin dependent regulator of chromatin, subfamily e, member 1 (SMARCE1), mRNA. | | | | | | | | | | |
| HOOK2 | 0.0306 | 0.1540188 |  | Homo sapiens hook homolog 2 (Drosophila) (HOOK2), mRNA. | | | | | |  |  |  |  |  |
| RILP | 0.0471 | 0.15337431 |  | Homo sapiens Rab interacting lysosomal protein (RILP), mRNA. | | | | | |  |  |  |  |  |
| JMJD1B | 0.0492 | 0.15243977 |  | Homo sapiens jumonji domain containing 1B (JMJD1B), mRNA. | | | | | |  |  |  |  |  |
| SLC17A8 | 0.0255 | 0.14997917 |  | Homo sapiens solute carrier family 17 (sodium-dependent inorganic phosphate cotransporter), member 8 (SLC17A8), mRNA. | | | | | | | | | | |
| KCNK10 | 0.0492 | 0.14883736 |  | Homo sapiens potassium channel, subfamily K, member 10 (KCNK10), transcript variant 2, mRNA. | | | | | | | | |  |  |
| ZFP37 | 0.0471 | 0.14721024 |  | Homo sapiens zinc finger protein 37 homolog (mouse) (ZFP37), mRNA. | | | | | | |  |  |  |  |
| ARMC4 | 0.0387 | 0.14676821 |  | Homo sapiens armadillo repeat containing 4 (ARMC4), mRNA. | | | | | |  |  |  |  |  |
| LOC441129 | 0.0332 | 0.14600976 |  | PREDICTED: Homo sapiens LOC441129 (LOC441129), mRNA. | | | | | |  |  |  |  |  |
| C1orf87 | 0.00809 | 0.14380394 |  | Homo sapiens chromosome 1 open reading frame 87 (C1orf87), mRNA. | | | | | | |  |  |  |  |
| LOC442669 | 0.0101 | 0.14379837 |  | PREDICTED: Homo sapiens similar to T-cell receptor gamma chain V region PT-gamma-1/2 precursor (LOC442669), mRNA. | | | | | | | | | | |
| HSPA2 | 0.0082 | 0.14104566 |  | Homo sapiens heat shock 70kDa protein 2 (HSPA2), mRNA. | | | | | |  |  |  |  |  |
| GPC3 | 0.029 | 0.13248526 |  | Homo sapiens glypican 3 (GPC3), mRNA. | | | |  |  |  |  |  |  |  |
| CYBRD1 | 0.0459 | 0.13223277 |  | Homo sapiens cytochrome b reductase 1 (CYBRD1), mRNA. | | | | | |  |  |  |  |  |
| LOC339951 | 0.0387 | 0.13104308 |  | PREDICTED: Homo sapiens similar to Cadherin-related tumor suppressor precursor (Fat protein) (LOC339951), mRNA. | | | | | | | | | | |
| TTYH2 | 0.0434 | 0.13089027 |  | Homo sapiens tweety homolog 2 (Drosophila) (TTYH2), transcript variant 1, mRNA. | | | | | | | |  |  |  |
| LOC441017 | 0.0374 | 0.12978986 |  | PREDICTED: Homo sapiens LOC441017 (LOC441017), mRNA. | | | | | |  |  |  |  |  |
| CAPN3 | 0.0303 | 0.12841031 |  | Homo sapiens calpain 3, (p94) (CAPN3), transcript variant 6, mRNA. | | | | | |  |  |  |  |  |
| COL3A1 | 0.0448 | 0.12613502 |  | Homo sapiens collagen, type III, alpha 1 (Ehlers-Danlos syndrome type IV, autosomal dominant) (COL3A1), mRNA. | | | | | | | | | |  |
| GMEB2 | 0.0255 | 0.12388435 |  | Homo sapiens glucocorticoid modulatory element binding protein 2 (GMEB2), mRNA. | | | | | | | |  |  |  |
| LOC401616 | 0.0455 | 0.1186523 |  | PREDICTED: Homo sapiens hypothetical LOC401616 (LOC401616), mRNA. | | | | | | |  |  |  |  |
| RBPMS2 | 0.0497 | 0.11785065 |  | Homo sapiens RNA binding protein with multiple splicing 2 (RBPMS2), mRNA. | | | | | | |  |  |  |  |
| LOC401980 | 0.0404 | 0.116373196 |  | PREDICTED: Homo sapiens similar to hypothetical protein 4933406M09 (LOC401980), mRNA. | | | | | | | | |  |  |
| LOC149620 | 0.0387 | 0.11243554 |  | Homo sapiens similar to CHIA protein (LOC149620), mRNA. | | | | | |  |  |  |  |  |
| PDIA4 | 0.0492 | 0.11206982 |  | Homo sapiens protein disulfide isomerase family A, member 4 (PDIA4), mRNA. | | | | | | |  |  |  |  |
| DNMBP | 0.0448 | 0.106690235 |  | - |  |  |  |  |  |  |  |  |  |  |
| CCL19 | 0.0259 | 0.104209244 |  | Homo sapiens chemokine (C-C motif) ligand 19 (CCL19), mRNA. | | | | | |  |  |  |  |  |
| LOC401188 | 0.0452 | 0.10237328 |  | PREDICTED: Homo sapiens hypothetical LOC401188 (LOC401188), mRNA. | | | | | | |  |  |  |  |
| LOC392443 | 0.029 | 0.100416906 |  | PREDICTED: Homo sapiens similar to ferritin light chain (LOC392443), mRNA. | | | | | | |  |  |  |  |
| MT2A | 0.0471 | 0.0996017 |  | - |  |  |  |  |  |  |  |  |  |  |
| AQP1 | 0.0258 | 0.097733796 |  | Homo sapiens aquaporin 1 (channel-forming integral protein, 28kDa) (AQP1), transcript variant 1, mRNA. | | | | | | | | | |  |
| POLR3A | 0.0485 | 0.09685922 |  | Homo sapiens polymerase (RNA) III (DNA directed) polypeptide A, 155kDa (POLR3A), mRNA. | | | | | | | |  |  |  |
| TF | 0.0452 | 0.09615042 |  | Homo sapiens transferrin (TF), mRNA. | | | |  |  |  |  |  |  |  |
| DSC3 | 0.029 | 0.091129564 |  | Homo sapiens desmocollin 3 (DSC3), transcript variant Dsc3b, mRNA. | | | | | | |  |  |  |  |
| ZNF554 | 0.0323 | 0.09092506 |  | Homo sapiens zinc finger protein 554 (ZNF554), mRNA. | | | | |  |  |  |  |  |  |
| HOXD3 | 0.044 | 0.08394394 |  | Homo sapiens homeo box D3 (HOXD3), mRNA. | | | | |  |  |  |  |  |  |
| DAOA | 0.028 | 0.083838776 |  | Homo sapiens D-amino acid oxidase activator (DAOA), mRNA. | | | | | |  |  |  |  |  |
| SPTLC2 | 0.00491 | 0.08334746 |  | Homo sapiens serine palmitoyltransferase, long chain base subunit 2 (SPTLC2), mRNA. | | | | | | | |  |  |  |
| FOXJ2 | 0.042 | 0.082834154 |  | Homo sapiens forkhead box J2 (FOXJ2), mRNA. | | | | |  |  |  |  |  |  |
| LOC440011 | 0.0408 | 0.080441676 |  | PREDICTED: Homo sapiens LOC440011 (LOC440011), mRNA. | | | | | |  |  |  |  |  |
| TMED7 | 0.0387 | 0.07774203 |  | Homo sapiens transmembrane emp24 protein transport domain containing 7 (TMED7), mRNA. | | | | | | | | |  |  |
| GAL | 0.0322 | 0.075857446 |  | Homo sapiens galanin (GAL), mRNA. | | | |  |  |  |  |  |  |  |
| BHMT2 | 0.00809 | 0.07052458 |  | Homo sapiens betaine-homocysteine methyltransferase 2 (BHMT2), mRNA. | | | | | | |  |  |  |  |
| SLC25A24 | 0.0288 | 0.069042124 |  | Homo sapiens solute carrier family 25 (mitochondrial carrier; phosphate carrier), member 24 (SLC25A24), transcript variant 1, mRNA. | | | | | | | | | | |
| KRT6L | 0.0452 | 0.06756804 |  | Homo sapiens keratin 6L (KRT6L), mRNA. | | | |  |  |  |  |  |  |  |
| C1orf73 | 0.029 | 0.066140525 |  | - |  |  |  |  |  |  |  |  |  |  |
| LOC389611 | 0.0342 | 0.06420752 |  | PREDICTED: Homo sapiens similar to FLJ10408 protein (LOC389611), mRNA. | | | | | | |  |  |  |  |
| NYD-SP26 | 0.0492 | 0.055969473 |  | Homo sapiens testis development protein NYD-SP26 (NYD-SP26), mRNA. | | | | | | |  |  |  |  |
| C16orf51 | 0.0387 | 0.054252595 |  | Homo sapiens chromosome 16 open reading frame 51 (C16orf51), mRNA. | | | | | | |  |  |  |  |
| LOC441513 | 0.0355 | 0.05332612 |  | PREDICTED: Homo sapiens LOC441513 (LOC441513), mRNA. | | | | | |  |  |  |  |  |
| KIAA0286 | 0.0343 | 0.045931578 |  | Homo sapiens KIAA0286 protein (KIAA0286), mRNA. | | | | |  |  |  |  |  |  |
| ADAM21 | 0.029 | 0.04409977 |  | Homo sapiens a disintegrin and metalloproteinase domain 21 (ADAM21), mRNA. | | | | | | | |  |  |  |
| HELB | 0.0276 | 0.042792775 |  | Homo sapiens helicase (DNA) B (HELB), mRNA. | | | | |  |  |  |  |  |  |
| OSTN | 0.0101 | 0.026838386 |  | Homo sapiens osteocrin (OSTN), mRNA. | | | |  |  |  |  |  |  |  |
