# Supplementary material for: Decreased SGK1 Expression and Function Contributes to Behavioral Deficits Induced by Traumatic Stress
Source: PLoS Biol. 2015 Oct 27;13(10):e1002282. doi: 10.1371/journal.pbio.1002282 (PMC4623974; doi:10.1371/journal.pbio.1002282)
Supplement: S3 Table — Healthy controls, n = 5; PTSD patients, n = 6. For the microarray, asterisk indicates significant p-value (*p < 0.05, FDR adjusted). Real-time qPCR for FGFR2 t(10) = 3.272, Student’s t test, **p < 0.01. (DOCX) [file pbio.1002282.s010.docx]

| Gene | Microarray | *P* value | qRT-PCR | *P* value |
| --- | --- | --- | --- | --- |
| MEF2C | upregulated | >0.05 | upregulated | >0.05 |
| Slc25A24 | downregulated | *<0.05 | downregulated | >0.05 |
| TLL1 | downregulated | >0.05 | downregulated | >0.05 |
| HTR7 | upregulated | >0.05 | upregulated | >0.05 |
| FGFR2 | downregulated | >0.05 | downregulated | **<0.01 |
